# Synthesis and Single Crystal Structures of Substituted-1,3-Selenazol-2-amines †

**DOI:** 10.3390/molecules22010046

**Published:** 2016-12-29

**Authors:** Guoxiong Hua, Junyi Du, Alexandra M. Z. Slawin, J. Derek Woollins

**Affiliations:** EaStCHEM School of Chemistry, University of St. Andrews, St. Andrews, Fife KY16 9ST, UK; gh15@st-andrews.ac.uk (G.H.); liangfengzhiyi@163.com (J.D.); amzs@st-andrews.ac.uk (A.M.Z.S.)

**Keywords:** cyanamides, haloketones, selenazol-2-amines, selenoureas, Woollins’ reagent

## Abstract

The synthesis and X-ray single crystal structures of a series of new 4-substituted-1,3-selenazol-2-amines is reported. The efficient preparation of these compounds was carried out by two-component cyclization of the selenoureas with equimolar amounts of α-haloketones. The selenoureas were obtained from the reaction of Woollins’ reagent with cyanamides, followed by hydrolysis with water. All new compounds have been characterized by IR spectroscopy, multi-NMR (^1^H, ^13^C, ^77^Se) spectroscopy, accurate mass measurement and single crystal X-ray structure analysis.

## 1. Introduction

Selenazoles have been extensively described as useful synthetic tools [1,2,3,4] with biologically significant antibiotic [5] and cancerostatic [6,7] and superoxide anion scavenging activity [8]. A few reports have appeared on the synthesis of selenazoles and thiazoles, including both solid phase [9] and solution phase synthesis [10,11,12]. Narender et al. reported the synthesis of selenazoles/thiazoles by the condensation of phenacylbromides/tosylates with selenourea/thiourea/thiobenzamide employing β-cyclodextrin as a catalyst [13,14]. Recently Varma and co-workers synthesized diaryl thiazoles from various α-tosyloxy ketones in water [15]. Several protocols are also described for the synthesis of thiazoles and selenazoles using promoters or catalysts in different organic solvents. However, development of novel environmentally benign approaches for the synthesis of selenazoles/thiazoles is highly desirable. The first ever tandem one-pot synthetic protocol for the synthesis of thiazoles/selenazoles from alkynes via the formation of 2,2-dibromo-1-phenylethanone has been reported. The reaction is catalyzed by β-cyclodextrin in aqueous medium and resulted in good yields [16]. A limitation to this route is the unavailability of the starting material primary selenamides for the preparation of the selenazoles. Many synthetic strategies to primary selenoamides have been documented, for example, by the reaction of nitrile with H_2_Se or NaSeH (generated in situ from NaBH_4_/Se) [17] or Se/CO [18,19,20,21] or P_2_Se_5_/H_2_O [22] and or tris(trimethylsilyl) monoselenophosphate [23]. In addition, although some alternative selenating reagents such as Al_2_Se_3_ [24], (Me_3_Si)_2_Se [25] and 4-methylselenobenzoate [26] have also been applied in these preparations, almost of these methods required prolonged reaction times, high temperature, and inconvenient reaction conditions or could not be reproduced [22]. We have previously reported a highly efficient approach for the preparation of a series of primary arylselenoamides from the reaction of arylnitriles with 2,4-bis(phenyl)-1,3-diselenadiphosphetane-2,4-diselenide [PhP(Se)(µ-Se)]_2_ (Woollins’ reagent) [27,28,29,30,31,32,33,34,35], followed by treatment of water [36]. By means of this privileged method, selenoureas might be prepared in excellent yields. Herein, we report a very facile route to prepare a series of novel 4-substituted-1,3-selenazol-2-amines and single crystal X-ray structural profiles of seven of the products.

## 2. Results and Discussion

**Scheme 1:**
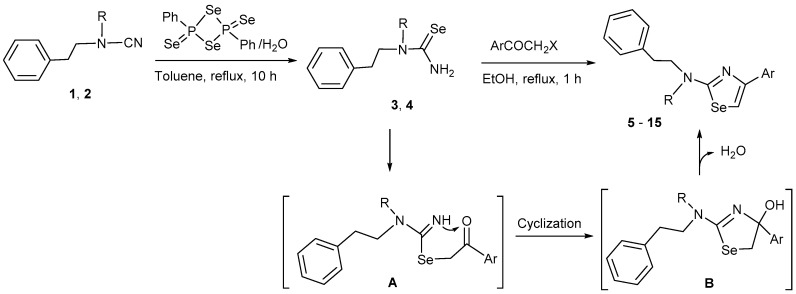
Synthesis of 4-substituted-1,3-selenazol-2-amines **5**–**15** (X, R and Ar groups defined in Table 1).

Cyanamides **1** and **2** were prepared in almost quantitative yields by the literature method from the reaction of cyanogen bromide with primary or secondary amines in dry methanol in the presence of excess of anhydrous CH_3_COONa at room temperature [37]. Two selenoureas **3** and **4** were obtained in the yields of 87% and 90%, respectively, by reaction of Woollins’s reagent with the corresponding cyanamides **1** and **2**, followed by post-treatment with water [38]. As shown in Scheme 1 and Table 1, cyclization of selenoureas **3** and **4** with an equivalent of the corresponding α-haloketones in refluxing ethanol solution gave a series of five-membered ring 4-substituted-1,3-selenazol-2-amines **5**–**15** in excellent yields. The scope of the reaction was expanded by the reaction of various selenoureas with phenylacetylene substrates and a variety of α-haloketones. In these reactions, substituents on the selenoureas did not have significant effect on the product yields. It is also interesting noting that electron-rich aryl rings allowed for cyclization reactions in yields comparable to electron-deficient aromatic moieties; and the steric hindrance was rarely permitted since the presence of CH_3_O, CH_3_, Cl, Br and NO_2_ groups in the 4-aryl ring had minimal to no effect on reaction yields.

The 4-substituted-1,3-selenazol-2-amines **5**–**15** are stable to air or moisture for months without any signs of degradation occurring. Characterization of 4-substituted-1,3-selenazol-2-amines **5**–**15** was performed by means of ^1^H-, ^13^C-, and ^77^Se-NMR, IR spectroscopy and mass spectrometry in conjunction with single crystal X-ray crystallography of seven of the compounds. All new compounds show the anticipated [M + H]^+^ peaks in their mass spectra, as well as satisfactory accurate mass measurements and appropriate isotopic distributions. The IR spectra show very strong bands ranging from 1554 to 1561 cm^−1^ for 4-substituted-1,3-selenazol-2-amines **5**–**12** and 1513 to 1517 cm^−1^ for 4-substituted-1,3-selenazol-2-amines **13**–**15**, attributed to the *ν*(N=C) vibration, accompanied by intense bands in the range 699 to 705 cm^−1^ being characteristic of the *ν*(C-Se) [39]. The CH_3_ group replaced by C_2_H_5_O(O)C group in amine N atom makes IR spectra of **2**, **4** and **13**–**15** for the *ν*(N=C) into higher frequency (ca. 40 cm^−1^). Furthermore, the ^1^H-NMR spectra exhibit the expected peaks including sharp singlet signals between 7.44 and 7.90 ppm assigned to the 1,3-selenazole rings. The ^13^C-NMR spectra have three signals typical for the 1,3-selenazole rings along with the expected signals from the aromatic carbon backbones (see Supplementary Materials). The ^77^Se-NMR spectra of all compounds **5**–**15** display singlet signals in the range 567.1–684.2 ppm, comparable to the signals of the related 2-dialkylamino-1,3-selenazoles (528.9–575.9 ppm) [40,41,42,43,44]; however, these values are significantly lower than that in 2,4-dialkyl- or 2,4-diaryl-1,3-selenazoles (657.8–767.1 ppm) [45,46,47] and 5-aminoselenazoles (629.0–707.0 ppm) [48]. The results indicated the high influence by the basic skeletons of selenazoles and the substituents close to the selenium atom [49]. It is worth noting that 4-substituted-1,3-selenazol-2-amines **13**–**15** bearing the electron-withdrawing substituted C_2_H_5_O(O)C group on the amine N atom center have much higher ^77^Se-NMR chemical shifts than 4-substituted-1,3-selenazol-2-amines **5**–**12** bearing the electron-donating substituted CH_3_ group on the amine N atom center.

The formation of 4-substituted-1,3-selenazol-2-amines **5**–**15** can be explained considering the reaction mechanism depicted in Scheme 1. The intermediate A, an addition product of selenoureas **3** or **4** and α-haloketones, undergoes a further cyclization reaction resulting in another intermediate B, which subsequently eliminates one molecule of H_2_O affording compounds **5**–**15**.

**Scheme 2:**
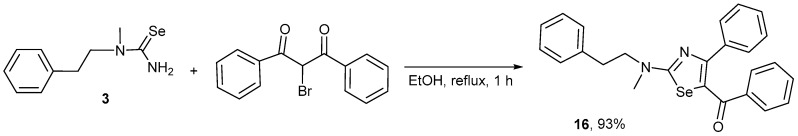
Synthesis of 4-phenyl-1,3-selenazol-5-yl)(phenyl)methanone **16**.

Similarly, treating selenourea **3** with an equivalent of 2-bromo-1,3-diphenylpropane-1,3-dione produced the corresponding 4-phenyl-1,3-selenazol-5-yl)(phenyl)methanone **16** in excellent yield (93%) as shown in Scheme 2. Compound **16** is a greyish yellow paste, soluble in common organic solvents. The anticipated [M + H]^+^ peak was observed in its mass spectra with satisfactory accurate mass measurement. No ^1^H-NMR signal was observed for the 1,3-selenazole ring except for the expected signals for the presence of phenyl rings. Not surprisingly, the ^77^Se-NMR spectrum comprises an expected sharp singlet at 609.7 ppm.

**Figure 1:**
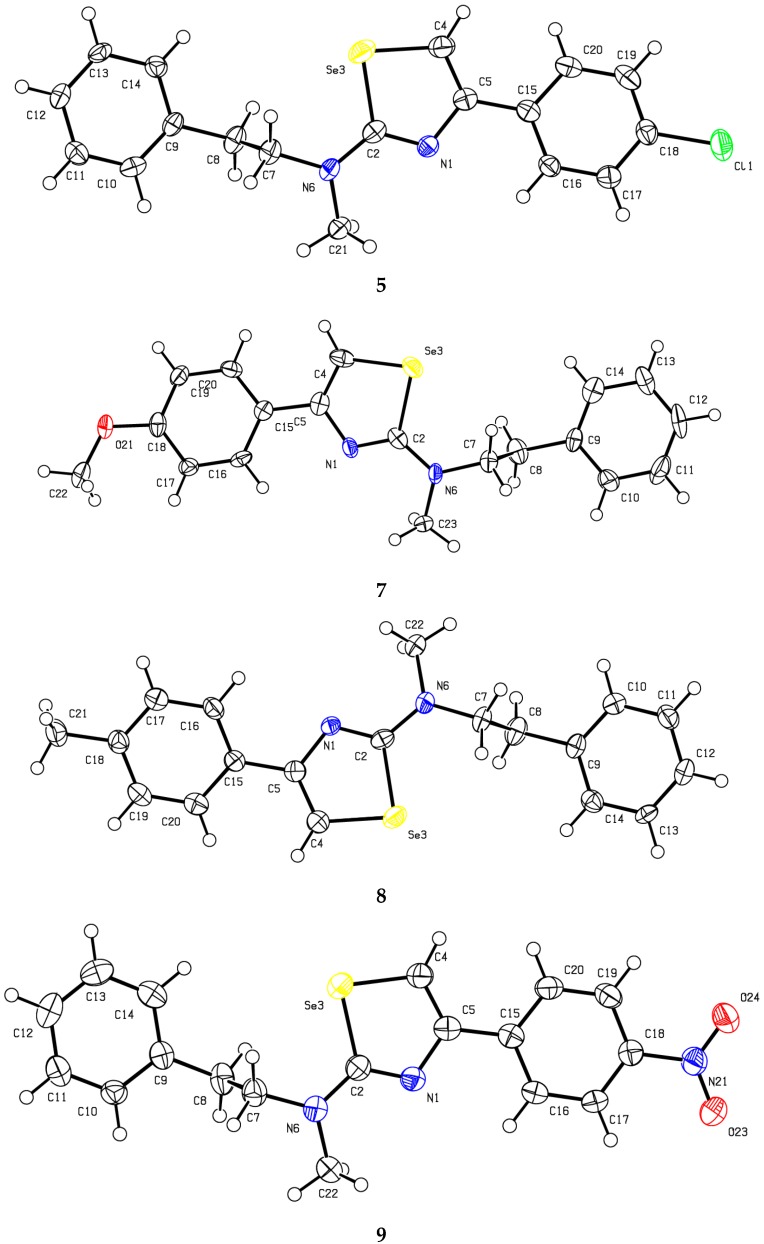

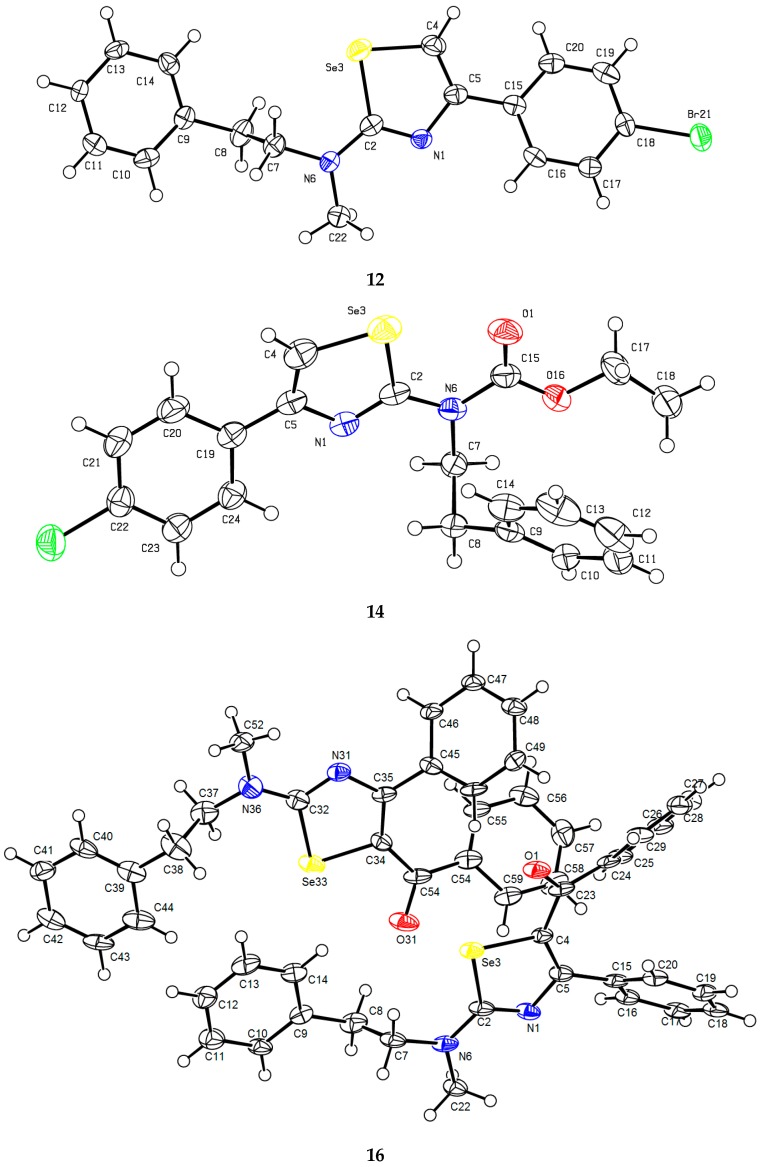
Single crystal X-ray structures of compound **5**, **7**, **8**, **9**, **12**, **14** and **16**.

Crystals of compounds **5**, **7**, **8**, **9**, **12**, **14** and **16** suitable for X-ray crystallographic analysis were grown by diffusion of a dichloromethane solution of the compound into hexane at room temperature in each case. The absolute structures of compounds **5**, **7**, **8**, **9**, **12**, **14** and **16** were determined using X-ray diffraction analysis as shown in Figure 1. Crystal data and structure refinement for compounds **5**, **7**, **8**, **9**, **12**, **14** and **16** are summarized in Table 2 and Table 3. Selected bond lengths and angles are listed in Table 4. All structures except **16** have a single molecule of the compound in the asymmetric unit and adopt very similar conformation; **16**, contains two independent molecules. In all cases, the newly formed 1,3-selenazole ring is not complete planar, and the mean plane of the newly formed five-membered ring is not coplanar with the adjacent aryl rings, with the dihedral angles of 21.61° in **5**, 17.98° in **7**, 22.78° in **8**, 8.04° in **9**, 21.59° in **12**, 18.99° in **14** and 47.14 [44.79]° in **16**. Two aryl rings (one is from the C_6_H_5_CH_2_CH_2_ group, another is the phenyl aryl ring attaching to the azole ring) are not parallel, with an angle 19.84° in **5**, 6.15° in **7**, 21.07° in **8**, 6.43° in **9**, 19.54° in **12**, 49.91° in **14** and 44.39 [34.21]° in **16**, the larger angles attribute to the effect of big substituted group [C_2_H_5_COC(O)] on N6 atom in **14** and an excess group [PhC(O)] on azole ring in **16**.

The bond lengths in **5**, **7**, **8**, **9**, **12**, **14** and **16** range from 1.286(12) to 1.329(12) Å for C2-N1 and 1.349(11) to 1.366(11) Å for C4-C5, respectively, which are comparable to that in the analogous structure of 2-piperidino-1,3-selenazole-5-carboxylic acid (1.330(3) and 1.359(4) Å, respectively) [22], indicating clearly their double bond character. The two C-N bond lengths of both C2-N6 (1.348(112) to 1.402(4) Å) and N1-C5 (1.374(11) to 1.401(4) Å) in **5**, **7**, **8**, **9**, **12**, **14** and **16** are marginally longer than that in 2-piperidino-1,3-selenazole-5-carboxylic acid (1.339(3) and 1.361(3) Å, respectively) [50], however, these values are significantly shorter than the usual single bond length of 1.47 Å [51]. The sums of the three angles around each of the C2 and C5 atoms are 360.0 and 359.81° in **5**, 359.95 and 359.89° in **7**, 359.99 and 359.78° in **8**, 360 and 359.98° in **9**, 359.99 and 359.88° in **12**, 360 and 359.99° in **14** and 359.76 [360]° and 359.91 [359.57]° in **16**, respectively. These results can be attributed to the delocalization of π-electrons and the lone pair electrons on N6. Also, it is worth noting that the N6 nitrogen has sp2 character rather than sp3 for all structures.

**Figure 2:**
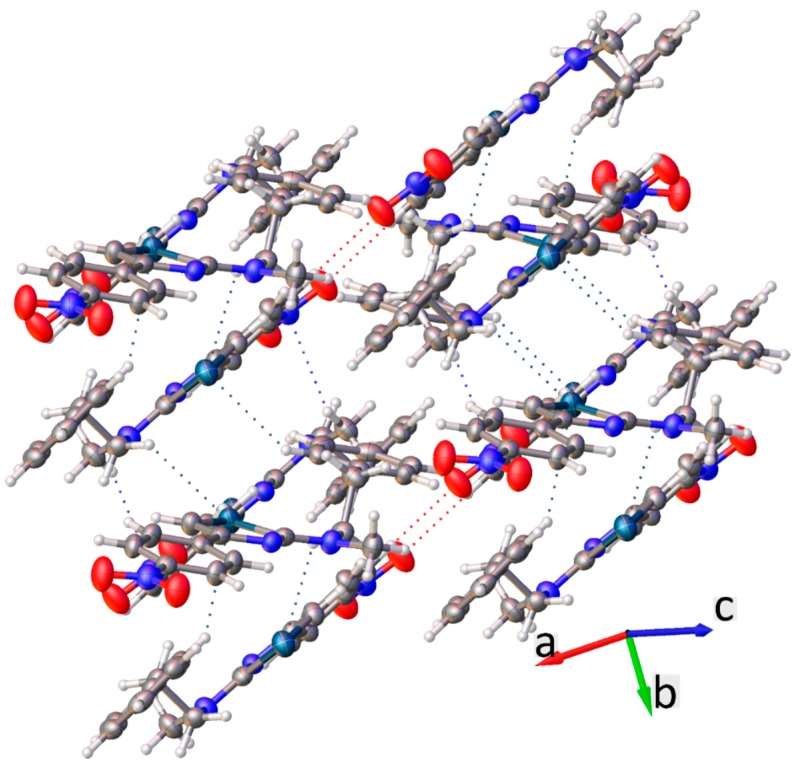
The packing framework shows the intermolecular C-H∙∙∙Se, C-H∙∙∙N and C-H∙∙∙O close contacts in **9**.

**Figure 3:**
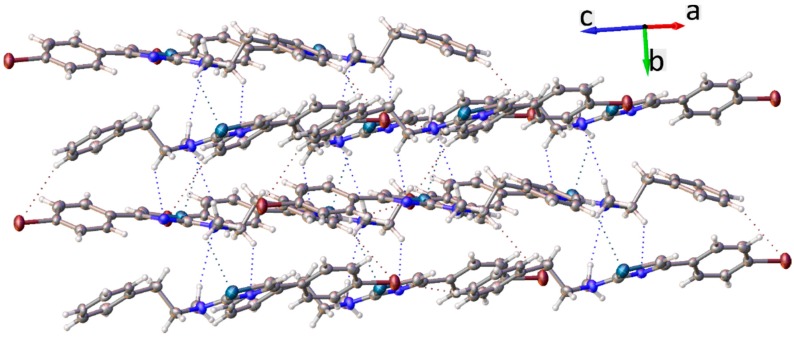
The packing framework shows the intermolecular C-H∙∙∙Se, C-H∙∙∙N and C-H∙∙∙Br close contacts in **12**.

Interestingly, in the supramolecular structures of **5**, **7**, **8**, **9**, **12**, **14** and **16**, no intramolecular close contacts were observed; however, a few intermolecular C-H∙∙∙Se, C-H∙∙∙N, C-H∙∙∙O, C-H∙∙∙Cl, C-H∙∙∙Br interactions are found (Figure 2 and Figure 3 as representative samples). In all structures, there have highly similar packing motifs with both selenium and nitrogen atoms within the azole ring involved in these close contacts. Furthermore, there is one or more intermolecular C-H∙∙∙O, C-H∙∙∙Cl and C-H∙∙∙Br close contacts in the structures of **5**, **7**, **9**, **12**, **14** and **16** apart from **8**, indicating that the presence of oxygen, chlorine, bromine and nitrogen atoms implicates these intermolecular close contacts.

## 3. Experimental Section

### 3.1. General Information

Unless otherwise stated, all reactions were carried out under on oxygen free nitrogen atmosphere using pre-dried solvents and standard Schlenk techniques, subsequent chromatographic and work up procedures were performed in air. ^1^H (400.1 MHz), ^13^C (100.6 MHz) and ^77^Se-{^1^H} (51.4 MHz referenced to external Me_2_Se) NMR spectra were recorded at 25 °C (unless stated otherwise) on Advance II 400s (Bruker, Blue Lion Biotech, Carnation, WA, USA) and GSX 270 (JEOL, Inc., Peabody, MA, USA) instrument. IR spectra were recorded as KBr pellets in the range of 4000–250 cm^−1^ on a 2000 FTIR/Raman spectrometer (Perkin-Elmer, Beaconsfield, UK). Mass spectrometry was performed by the EPSRC National Mass Spectrometry Service Centre, Swansea. X-ray crystal data for compounds **5**, **7**, **8**, **9**, **12**, **14** and **16** were collected using a SCXMIni Mercury CCD system (Rigaku, Houston, USA). Intensity data were collected using ω steps accumulating area detector images spanning at least a hemisphere of reciprocal space. All data were corrected for Lorentz polarization effects. Absorption effects were corrected based on multiple equivalent reflections or by semi-empirical methods. Structures were solved by direct methods and refined by full-matrix least-squares against F^2^ by using the program SHELXTL [52]. Hydrogen atoms were assigned riding isotropic displacement parameters and constrained to idealized geometries. These data (CCDC 1522917–1522923) can be obtained free of charge via www.ccdc.cam.ac.uk/conts/retrieving.html or from the Cambridge Crystallographic Data center, 12 Union Road, Cambridge CB2 1EZ, UK; fax: +44-1223-336-033; e-mail: deposit@ccdc.cam.ac.uk.

### 3.2. Synthesis

#### General Procedure for the Synthesis of Compounds **5**–**16**

A mixture of α-haloketone (1.0 mmol) in dry methanol (10 mL) was added dropwise to a refluxing solution of arylselenocarboamide (1.0 mmol) in dry methanol (20 mL) over the course of 1 h. The reaction mixture was then refluxed for another 1 h. After cooling to room temperature, the mixture was concentrated on a rotary evaporator, and the residue was neutralized with 5% aqueous ammonia (30 mL), extracted with dichloromethane (30 mL × 3), and the combined organic layers washed with water (20 mL × 3), brine (20 mL), and dried over MgSO_4_. After filtering and drying to remove the solvent the organic residue was purified by silica gel column chromatography (1:9 ethyl acetate/dichloromethane as eluent) to give 1,3-selenazoles **5**–**16**.

*N-Methyl-N-phenethyl-4-phenyl-1,3-selenazol-2-amine* (**5**). Pale yellow paste (0.315 g, 92%). Selected IR (KBr, cm^−1^): 1555, 1480, 1453, 1362, 1324, 1299, 1171, 1099, 1043, 936, 772, 748, 699, 564, 496. ^1^H-NMR (CD_2_Cl_2_, δ), 7.90 (s, 1H), 7.88 (d, *J*(H,H) = 8.3 Hz, 2H), 7.40–7.21 (m, 8H), 3.73 (t, *J*(H,H) = 7.4 Hz, 2H), 3.05 (s, 3H), 3.01 (t, *J*(H,H) = 7.4 Hz, 2H) ppm. ^13^C-NMR (CD_2_Cl_2_, δ), 171.2, 152.9, 139.3, 136.2), 13.0, 129.0, 128.6, 128.5, 127.3, 126.4, 104.8, 56.0, 39.8, 33.4 ppm. ^77^Se-NMR (CD_2_Cl_2_, δ), 575.3 ppm. HRMS (CI^+^, *m*/*z*): found 343.0717 [M + H]^+^, calculated mass for C_18_H_18_N_2_SeH: 343.0713.

*4-(4-Chlorophenyl)-N-methyl-N-phenethyl-1,3-selenazol-2-amine* (**6**). Pale white solid (0.362 g, 96%). M.p. 82–84 °C. Selected IR (KBr, cm^−1^): 1554, 1457, 1396, 1363, 1317, 1264, 1175, 1086, 1040, 1009, 935, 838, 756, 703, 679, 496. ^1^H-NMR (CD_2_Cl_2_, δ), 7.83 (d, *J*(H,H) = 8.5 Hz, 2H), 7.81 (s, 1H), 7.35–7.21 (m, 7H), 3.72 (t, *J*(H,H) = 7.7 Hz, 2H), 3.03 (s, 3H), 3.00 (t, *J*(H,H) = 7.7 Hz, 2H) ppm. ^13^C-NMR (CD_2_Cl_2_, δ), 171.3, 151.7, 139.2, 134.8, 132.7, 128.9, 128.6, 128.5, 127.7, 126.4, 105.3, 56.0, 39.8, 33.3 ppm. ^77^Se-NMR (CD_2_Cl_2_, δ), 571.1 ppm. HRMS (ES^+^, *m*/*z*): found 377.0321 [M + H]^+^, calculated mass for C_18_H_17_N_2_ClSeH: 377.0324.

*4-(4-Methoxyphenyl)-N-methyl-N-phenethyl-1,3-selenazol-2-amine* (**7**). Dark yellow solid (0.360 g, 97%). M.p. 74–76 °C. Selected IR (KBr, cm^−1^): 1560, 1490, 1455, 1455, 1408, 1357, 1320, 1244, 1170, 1107, 1029, 934, 834, 751, 703, 601, 499. ^1^H-NMR (CD_2_Cl_2_, δ), 7.83 (s, 1H), 7.80 (d, *J*(H,H) = 8.3 Hz, 2H), 7.34–7.15 (m, 5H), 6.88 (d, *J*(H,H) = 8.3 Hz, 2H), 3.82 (s, 3H), 3.71 (t, *J*(H,H) = 7.7 Hz, 2H), 3.05 (s, 3H), 3.00 (t, *J*(H,H) = 7.7 Hz, 2H) ppm. ^13^C-NMR (CD_2_Cl_2_, δ), 171.1, 159.1, 152.6, 139.3, 129.2, 129.0, 128.6, 127.6, 126.4, 113.7, 102.8, 56.0, 55.3, 40.0, 33.4 ppm. ^77^Se-NMR (CD_2_Cl_2_, δ), 567.1 ppm. HRMS (CI^+^, *m*/*z*): found 373.0811 [M + H]^+^, calculated mass for C_19_H_20_N_2_OSeH: 373.0814.

*4-(4-Methylphenyl)-N-methyl-N-phenethyl-1,3-selenazol-2-amine* (**8**). Yellow solid (0.350 g, 98%). M.p. 90–91 °C. Selected IR (KBr, cm^−1^): 1561, 1487, 1457, 1406, 1363, 1321, 1265, 1173, 1110, 1040, 1018, 934, 826, 754, 702, 673, 600, 502. ^1^H-NMR (CD_2_Cl_2_, δ), 7.76 (d, *J*(H,H) = 8.2 Hz, 2H), 7.35–7.17 (m, 8H), 3.73 (t, *J*(H,H) = 7.4 Hz, 2H), 3.05 (s, 3H), 3.01 (t, *J*(H,H) = 7.4 Hz, 2H), 2.35 (s, 3H) ppm. ^13^C-NMR (CD_2_Cl_2_, δ), 171.1, 153.0, 139.3, 137.2, 133.5, 129.1, 129.0, 128.6, 126.4, 126.2, 103.9, 56.0, 39.8, 33.4, 21.0 ppm. ^77^Se-NMR (CD_2_Cl_2_, δ), 568.1 ppm. HRMS (CI^+^, *m*/*z*): found 357.0867 [M + H]^+^, calculated mass for C_19_H_10_N_2_SeH: 357.0865.

*N-Methyl-4-(4-nitrophenyl)-N-phenethyl-1,3-selenazol-2-amine* (**9**). Yellow solid (0.360 g, 93%). M.p. 96–98 °C. Selected IR (KBr, cm^−1^): 1600, 1593, 1558, 1501, 1406, 1336, 1174, 1106, 1045, 935, 857, 847, 755, 703, 501. ^1^H-NMR (CDCl_3_, δ), 8.15 (d, *J*(H,H) = 9.0 Hz, 2H), 7.94 (d, *J*(H,H) = 9.0 Hz, 2H), 7.44 (s, 1H), 7.27–7.17 (m, 5H), 3.68 (t, *J*(H,H) = 7.7 Hz, 2H), 3.00 (s, 3H), 2.95 (t, *J*(H,H) = 7.7 Hz, 2H) ppm. ^13^C-NMR (CDCl_3_, δ), 171.4, 151.0, 146.6, 141.9, 138.8, 128.9, 128.7, 126.8, 126.6, 124.0, 109.1, 56.2, 40.1, 33.4 ppm. ^77^Se-NMR (CDCl_3_, δ), 590.1 ppm. HRMS (CI^+^, *m*/*z*): found 388.0557 [M + H]^+^, calculated mass for C_18_H_17_N_3_O_2_SeH: 388.0559.

*4-(2,5-Dimethoxyphenyl)-N-methyl-N-phenethyl-1,3-selenazol-2-amine* (**10**). Green oil (0.360 g, 90%). Selected IR (KBr, cm^−1^): 1674, 1558, 1496, 1464, 1409, 1357, 1280, 1217, 1178, 1047, 1023, 809, 744, 700, 585. ^1^H-NMR (CDCl_3_, δ), 7.76 (s, 1H, Azole-H), 7.21–7.13 (m, 5H, Ar-H), 6.94 (d, *J*(H,H) = 7.7 Hz, 1H, Ar-H), 6.82 (s, 1H), 6.79 (d, *J*(H,H) = 7.7 Hz, 1H), 3.80 (s, 3H), 3.70 (s, 3H), 3.63 (t, *J*(H,H) = 7.7 Hz, 2H), 2.96 (s, 3H), 2.93 (t, *J*(H,H) = 7.7 Hz, 2H) ppm. ^13^C NMR (CDCl_3_, δ), 169.2, 153.6, 139.2, 128.9, 128.6, 128.3, 126.4, 120.4, 116.1, 113.8, 113.2, 112.5, 110.7, 56.0, 55.8, 55.7, 40.0, 33.4 ppm. ^77^Se-NMR (CDCl_3_, δ), 572.7 ppm. HRMS (CI^+^, *m*/*z*): found 403.0915 [M + H]^+^, calculated mass for C_20_H_22_N_2_O_2_SeH: 403.0919.

*4-(2,4-Dichlorophenyl)-N-methyl-N-phenethyl-1,3-selenazol-2-amine* (**11**). Yellow oil (0.375 g, 91%). Selected IR (KBr, cm^−1^): 1697, 1560, 1550, 1496, 1464, 1370, 1309, 1172, 1100, 1030, 936, 866, 825, 797, 747, 699, 554, 529, 497. ^1^H-NMR (CDCl_3_, δ), 7.78 (d, *J*(H,H) = 8.5 Hz, 2H), 7.45 (s, 1H), 7.44 (d, *J*(H,H) = 8.4 Hz, 1H), 7.36–7.35 (m, 2H), 7.22–7.15 (m, 3H), 3.62 (t, *J*(H,H) = 7.7 Hz, 2H), 2.94 (s, 3H), 2.93 (t, *J*(H,H) = 7.7 Hz, 2H) ppm. ^13^C-NMR (CDCl_3_, δ), 169.2, 147.6, 137.9, 132.5, 132.0, 131.5, 129.7, 129.0, 127.8, 127.6, 125.9, 125.4, 109.5, 55.1, 39.0, 32.4 ppm. ^77^Se-NMR (CDCl_3_, δ), 578.9 ppm. HRMS (CI^+^, *m*/*z*): found 410.9921 [M + H]^+^, calculated mass for C_18_H_16_Cl_2_N_2_SeH: 410.9924.

*4-(4-Bromophenyl)-N-methyl-N-phenethyl-1,3-selenazol-2-amine* (**12**). Yellow solid (0.418 g, 95%). Selected IR (KBr, cm^−1^): 1559, 1472, 1455, 1407, 1392, 1367, 1318, 1176, 1069, 1007, 937, 836, 753, 705, 676, 491. ^1^H-NMR (CDCl_3_, δ), 7.67 (d, *J*(H,H) = 8.7 Hz, 2H), 7.59 (s, 1H), 7.40 (d, *J*(H,H) = 8.7 Hz, 2H), 7.26–7.15 (m, 5H), 3.65 (t, *J*(H,H) = 7.5 Hz, 2H), 2.97 (s, 3H), 2.93 (t, *J*(H,H) = 7.5 Hz, 2H) ppm. ^13^C-NMR (CDCl_3_, δ), 171.2, 152.0, 139.0, 135.1, 131.5, 128.9, 128.7, 128.0, 126.5, 125.4, 105.3, 56.2, 40.0, 29.7 ppm. ^77^Se-NMR (CDCl_3_, δ), 577.8 ppm. HRMS (CI^+^, *m*/*z*): found 420.9809 [M + H]^+^, calculated mass for C_18_H_17_BrN_2_SeH: 420.9811.

*Ethyl phenethyl(4-phenyl-1,3-selenazol-2-yl)carbamate* (**13**). Yellowish white solid (0.384 g, 96%). M.p. 65–67 °C. Selected IR (KBr, cm^−1^): 1695, 1600, 1517, 1477, 1439, 1408, 1383, 1269, 1197, 1025, 880, 753, 718, 699, 666, 499. ^1^H-NMR (CD_2_Cl_2_, δ), 7.95 (d, *J*(H,H) = 8.5 Hz, 2H), 7.79 (s, 1H), 7.44–7.29 (m, 8H), 4.44 (q, *J*(H,H) = 7.2 Hz, 2H), 4.21 (t, *J*(H,H) = 6.9 Hz, 2H), 3.08 (t, *J*(H,H) = 6.9 Hz, 2H), 1.29 (t, *J*(H,H) = 7.2 Hz, 3H) ppm. ^13^C-NMR (CD_2_Cl_2_, δ), 161.8, 150.6, 139.1, 136.0, 129.1, 128.6, 128.5, 127.55, 126.4, 126.2, 113.3 ppm. ^77^Se-NMR (CD_2_Cl_2_, δ), 679.9 ppm. HRMS (ES^+^, *m*/*z*): found 401.0766 [M + H]^+^, calculated mass for C_20_H_20_N_2_O_2_SeH: 401.0768.

*Ethyl (4-(4-chlorophenyl)-1,3-selenazol-2-yl)(phenethyl)carbamate* (**14**). Pale orange solid (0.416 g, 96%). M.p. 68–70 °C. Selected IR (KBr, cm^−1^): 1698, 1518, 1474 1441, 1382, 1314, 1244, 1189, 1089, 1030, 839, 739, 701, 578, 554, 498. ^1^H-NMR (CD_2_Cl_2_, δ), 7.89 (d, *J*(H,H) = 8.5 Hz, 2H), 7.77 (s, 1H), 7.39 (d, *J*(H,H) = 8.5 Hz, 2H), 7.31–7.22 (m, 5H), 4.42 (t, *J*(H,H) = 6.9 Hz, 2H), 4.19 (q, *J*(H,H) = 7.2 Hz, 2H), 3.07 (d, *J*(H,H) = 7.2 Hz, 2H), 1.29 (t, *J*(H,H) = 6.9 Hz, 3H) ppm. ^13^C-NMR (CD_2_Cl_2_, δ), 162.0, 149.4, 139.0, 134.6, 133.0, 129.1, 128.7, 128.5, 127.6, 126.5, 113.9, 63.4, 48.5, 34.2, 14.2 ppm. ^77^Se-NMR (CD_2_Cl_2_, δ), 684.2 ppm. HRMS (CI^+^, *m*/*z*): found 435.0375 [M + H]^+^, calculated mass for C_20_H_19_N_2_ClO_2_SeH: 435.0378.

*Ethyl (4-(4-methoxyphenyl)-1,3-selenazol-2-yl)(phenethyl)carbamate* (**15**). Pale yellow solid (0.410 g, 95%). M.p. 52–54 °C. Selected IR (KBr, cm^−1^): 1689, 1603, 1578, 1513, 1438, 1405, 1382, 320, 1301, 1250, 1176, 1027, 881, 835, 748, 700, 618, 562. ^1^H-NMR (CD_2_Cl_2_, δ), 7.87 (d, *J*(H,H) = 8.8 Hz, 2H), 7.62 (s, 1H), 7.31–7.25 (m, 5H), 6.94 (d, *J*(H,H) = 8.0 Hz, 2H), 4.43 (t, *J*(H,H) = 6.9 Hz, 2H), 4.20 (q, *J*(H,H) = 6.6 Hz, 2H), 3.83 (s, 3H), 3.07 (d, *J*(H,H) = 6.9 Hz, 2H), 1.29 (t, *J*(H,H) = 6.6 Hz, 3H) ppm. ^13^C-NMR (CD_2_Cl_2_, δ), 161.6, 159.3, 150.4, 139.1, 130.5, 129.1, 128.5, 127.4, 126.4, 113.9, 113.6, 111.2, 63.3, 55.3, 48.5, 34.2, 14.2 ppm. ^77^Se-NMR (CD_2_Cl_2_, δ), 675.7 ppm. HRMS (CI^+^, *m*/*z*): found 431.0867 [M + H]^+^, calculated mass for C_21_H_22_N_2_O_3_SeH: 431.0870.

*(2-(Methyl(phenethyl)amino)-4-phenyl-1,3-selenazol-5-yl)(phenyl)methanone* (**16**). Pale yellow paste (0.415 g, 93%). Selected IR (KBr, cm^−1^): 1595, 1575, 1542, 1473, 1327, 1284, 1103, 1025, 881, 779, 697, 670, 599. ^1^H-NMR (CDCl_3_, δ), 7.35–7.32 (m, 2H), 7.27–7.22 (m, 4H), 7.19–7.16 (m, 3H), 7.13–7.09 (m, 2H), 7.04–6.93 (m, 4H), 4.04 (t, *J*(H,H) = 7.4 Hz, 2H), 2.97 (t, *J*(H,H) = 7.4 Hz, 2H), 1.97 (s, 3H) ppm. ^13^C-NMR (CDCl_3_, δ), 190.3, 172.9, 160.6, 138.5, 138.4, 136.0, 131.9, 130.1, 129.3, 129.1, 128.7, 127.5, 127.4, 126.7, 60.4, 33.5, 15.0 ppm. ^77^Se-NMR (CDCl_3_, δ), 609.7 ppm. HRMS (CI^+^, *m*/*z*): found 447.0968 [M + H]^+^, calculated mass for C_25_H_22_N_2_OSeH: 447.0972.

## 4. Conclusions

In summary, a series of new 4-substituted-1,3-selenazol-2-amines were prepared in excellent yields by two-component cyclization of α-haloketones with equimolar amounts of selenoureas which were obtained from the reaction of Woollins’ reagent with cyanamides, followed by hydrolysis. The structures of all new compounds have been elucidated by using ^1^H-, ^13^C-, ^77^Se-NMR spectroscopy and accurate mass measurements. Seven single crystal X-ray structures reveal slightly different structure profiles. In all cases, the newly formed 1,3-selenazole ring is not complete planar, and none of the mean planes of the newly formed five-membered ring are coplanar with the adjacent aryl rings, showing different dihedral angles. Interestingly, no intramolecular close contacts were found; however, intermolecular C-H∙∙∙Se, C-H∙∙∙N, C-H∙∙∙O, C-H∙∙∙Cl and C-H∙∙∙Br short interactions are found in the structures and the oxygen, chlorine, bromine and nitrogen atoms play very key roles in these intermolecular close contacts.

## Figures and Tables

**Table 1:** Definition of the X, R and Ar Groups, Yields and ^77^Se-NMR Data for Compounds **5**–**15**.

Compound	X	R	Ar	Yield (%)	^77^Se-NMR (δ, ppm)
**1**	-	CH_3_	-	99	-
**2**	-	C_2_H_5_O(O)C	-	99	-
**3**	-	CH_3_	-	87	607.7
**4**	-	C_2_H_5_O(O)C	-	90	382.1
**5**	Cl	CH_3_	C_6_H_5_	92	575.3
**6**	Br	CH_3_	4-ClC_6_H_4_	96	571.1
**7**	Br	CH_3_	4-MeOC_6_H_4_	97	567.1
**8**	Br	CH_3_	4-MeC_6_H_4_	98	568.1
**9**	Br	CH_3_	4-NO_2_C_6_H_4_	93	590.1
**10**	Br	CH_3_	2,5-di-MeOC_6_H_3_	90	572.7
**11**	Cl	CH_3_	2,4-di-ClC_6_H_3_	91	578.9
**12**	Br	CH_3_	4-BrC_6_H_4_	95	577.8
**13**	Cl	C_2_H_5_O(O)C	C_6_H_5_	96	679.9
**14**	Br	C_2_H_5_O(O)C	4-ClC_6_H_4_	96	684.2
**15**	Br	C_2_H_5_O(O)C	4-MeOC_6_H_4_	95	675.7

**Table 2:** Details of the X-ray Data Collections and Refinements for Compounds **5**, **7**, **8** and **9**.

	Compound			
	5	7	8	9
Formula	C_18_H_17_ClN_2_Se	C_19_H_20_N_2_OSe	C_19_H_20_N_2_Se	C_18_H_17_N_3_O_2_Se
*M*	375.76	371.34	355.34	386.31
Crystal system	monoclinic	orthorhombic	monoclinic	monoclinic
Space group	P2_1_	P2_1_2_1_2_1_	P2_1_	P2_1_/n
a/Å	10.657(7)	6.6544(8)	10.719(4)	10.5159(7)
b/Å	7.525(5)	7.8134(9)	7.495(3)	7.5401(5)
c/Å	11.320(8)	33.372(4)	11.294(4)	20.9330(15)
α	90	90	90	90
β	115.852(8)	90	115.650(6)	91.879(2)
γ	90	90	90	90
U/A^3^	817.0(10)	1735.1(4)	818.0(5)	1658.9(2)
Z	2	4	2	4
µ/cm^−1^	24.591	21.703	22.939	22.793
Reflections collected	7051	11,930	6175	12,160
Independent reflections	2587	3043	1540	2895
R_int_	0.0291	0.1535	0.0336	0.0679
R1	0.0249	0.0695	0.0532	0.0394
wR2 [I > 2σ(I)]	0.0544	0.1012	0.1398	0.0903

**Table 3:** Details of the X-ray Data Collections and Refinements for Compounds **12**, **14** and **16**.

	Compound		
	12	14	16
Formula	C_18_H_17_BrN_2_Se	C_20_H_19_ClN_2_O_2_Se	C_25_H_22_N_2_OSe
*M*	420.21	433.80	445.42
Crystal system	monoclinic	orthorhombic	monoclinic
Space group	P2_1_	Pbca	P2_1_/c
a/Å	10.6448(10)	27.974(16)	10.1342(7)
b/Å	7.4660(7)	17.910(11)	14.6025(10)
c/Å	11.5064(11)	7.817(5)	27.942(2)
α	90	90	90
β	116.039(3)	90	90.421(4)
γ	90	90	90
U/A^3^	821.64(14)	3916(4)	4134.9(5)
Z	2	8	8
µ/cm^−1^	47.219	20.701	18.350
Reflections collected	6350	26,522	31,646
Independent reflections	2839	3437	7262
R_int_	0.0536	0.0694	0.2391
R1	0.0309	0.0422	0.0701
wR2 [I > 2σ(I)]	0.0639	0.0975	0.1456

**Table 4:** Selected Bond Distances (Å) and Angles (°) for Compounds **5**, **7**, **8**, **9**, **12**, **14** and **16**.

	5	7	8	9	12	14	16
N1-C2	1.302(5)	1.295(11)	1.300(13)	1.291(4)	1.300(9)	1.301(4)	1.329(12)[1.286(12)]
C2-N6	1.358(5)	1.350(11)	1.348 (12)	1.350(5)	1.368(9)	1.402(4)	1.348(12)[1.369(12)]
C2-Se3	1.906(3)	1.924(8)	1.914(7)	1.912(3)	1.896(5)	1.899(3)	1.863(8)[1.886(7)]
Se3-C4	1.860(5)	1.886(9)	1.858(12)	1.854(4)	1.862(8)	1.872(4)	1.866(8)[1.898(9)]
C4-C5	1.349(5)	1.353(12)	1.355(12)	1.359(5)	1.358(8)	1.356(5)	1.366(11)[1.349(11)]
C5-N1	1.394(4)	1.395(11)	1.387(10)	1.389(4)	1.387(7)	1.401(4)	1.374(11)[1.378(11)]
N1-C2-N6	123.8(3)	125.1(8)	124.5(7)	124.8(3)	123.2(5)	120.3(3)	120.5(7)[121.5(7)]
N1-C2-Se3	115.1(3)	114.5(6)	114.4(6)	114.9(3)	115.7(4)	116.1(2)	115.6(6)[117.1(6)]
Se3-C2-N6	121.1(3)	120.3(6)	121.1(7)	120.3(2)	121.1(5)	123.7(2)	123.8(7)[121.4(6)]
C2-Se3-C4	83.59(18)	83.9(4)	83.6(4)	83.370(15)	83.1(3)	83.10(15)	84.3(4)[82.5(4)]
Se3-C4-C5	111.5(3)	110.1(6)	111.6(7)	111.3(3)	111.8(5)	111.9(3)	110.6(6)[110.2(6)]
C5-N1-C2	112.0(3)	112.7(7)	112.9(6)	112.6(3)	111.6(4)	112.0(3)	111.7(7)[111.6(7)]
N1-C5-C15	117.1(3)	117.2(7)	118.2(7)	117.5(3)	117.3(4)	116.3(3) *	112.9(7)[113.7(7)]
N1-C5-C4	117.8(4)	118.8(8)	117.4(9)	117.5(3)	117.7(6)	116.9(3)	117.8(8)[118.4(8)]
C4-C5-C15	124.9(3)	123.9(8)	124.2(8)	124.9(3)	124.9(6)	126.8(3) *	129.2(8)[127.6(8)]

* C15 should be C19 in compound **14**.

## Supplementary Materials

Supplementary materials can be accessed at: http://www.mdpi.com/1420-3049/22/1/46/s1.

## References

[1] Casar Z., Majcen-Le Marechal A., Lorey D. (2003). A novel approach to a substituted 1,3-selenazole core as a precursor of electron rich olefins: Diselenadiazafulvalene and azino-diselenadiazafulvalene. New J. Chem..

[2] Koketsu M., Ishihara H. (2003). Synthesis of 1,3-selenazine and 1,3-selenazole and their biological activities. Curr. Org. Chem..

[3] Duddeck H., Bradenahl R., Stefaniak L., Jazwinski J., Kamienski B. (2001). Synthesis and multinuclear magnetic resonance investigation of some 1,3-selenazole and 1,3-selenazoline derivatives. Magn. Reson. Chem..

[4] Archer S., McGarry R. (1982). Diazotization of a 2-amino-1,3-selenazole. J. Heterocycl. Chem..

[5] Koketsu M., Choi S.Y., Ishihara H., Lim B.O., Kim H., Kim S.Y. (2002). Inhibitory effects of 1,3-selenazol-4-one derivatives on mushroom tyrosinase. Chem. Pharm. Bull..

[6] Goldstein B.M., Kennedy S.D., Hennen W.J. (1990). Selenium-77 NMR and crystallographic studies of selenazofurin and its 5-amino derivative. J. Am. Chem. Soc..

[7] Shafiee A., Shafaati A., Khamench B.H. (1989). Selenium heterocycles. XXXIX. Synthesis of thieno[3,4-*d*]thiazole, thieno[3,4-*d*]selenazole, selenolo[3,4-*d*]thiazole and selenolo[3,4-*d*]selenazole. J. Heterocycl. Chem..

[8] Sekhiguchi A., Nishina A., Kimura H., Fukumoto R.H., Koichi K., Ishihara H., Koketsu M. (2005). Superoxide anion-scavenging effect of 2-amino-1,3-selenazoles. Chem. Pharm. Bull..

[9] Kazzouli S.E., Raboin S.B., Mouadbib A., Guillaumet G. (2002). Solid support synthesis of 2,4-disubstituted thiazoles and aminothiazoles. Tetrahedron Lett..

[10] Bailey N., Dean A.W., Judd D.B., Middlemiss D., Storer R., Stephen P.W. (1996). A convenient procedure for solution phase preparation of 2-aminothiazole combinatorial libraries. Bioorg. Med. Chem. Lett..

[11] Kearney P.C., Fernandez M., Flygare J.A. (1998). Solid-phase synthesis of 2-aminothiazoles. J. Org. Chem..

[12] Goff D., Fernandez J. (1999). The preparation of 2,4-disubstituted thiazoles on solid support. Tetrahedron Lett..

[13] Narender M., Somi Reddy M., Kumar V.P., Reddy V.P., Nageswar Y.V.D., Rao K.R. (2007). Supramolecular synthesis of selenazoles using selenourea in water in the presence of β-cyclodextrin under atmospheric pressure. J. Org. Chem..

[14] Narender M., Somi Reddy M., Sridhar R., Nageswar Y.V.D., Rao K.R. (2005). Aqueous phase synthesis of thiazoles and aminothiazoles in the presence of β-cyclodextrin. Tetrahedron Lett..

[15] Dalip K., Kumar N.M., Patel G., Gupta S., Varma R.S. (2011). A facile and eco-friendly synthesis of diarylthiazoles and diarylimidazoles is described utilizing a facile reaction of α-tosyloxyketones in water. Tetrahedron Lett..

[16] Madhav B., Narayana Murthy S., Anil Kumar B.S.P., Ramesh K., Nageswar Y.V.D. (2012). A tandem one-pot aqueous phase synthesis of thiazoles/selenazoles. Tetrahedron Lett..

[17] Klayman D.L., Griffins T.S. (1973). Reaction of selenium with sodium borohydride in protic solvents. A facile method for the introduction of selenium into organic molecules. J. Am. Chem. Soc..

[18] Lai L.L., Reid D.H. (1993). Synthesis of primary selenocarboxamides and conversion of alkyl selenocarboxamides into selenazoles. Synthesis.

[19] Koketsu M., Fukuta Y., Nada F. (2001). Reaction of lithium aluminum hydride with elemental selenium: Its application as a selenating reagent into organic molecules. J. Am. Chem. Soc..

[20] Koketsu M., Fukuta Y., Ishihara H. (2001). Preparation of *N*,*N*-unsubstituted selenoureas and thioureas from cyanamides. Tetrahedron Lett..

[21] Ogawa A., Miyaka J., Karasaki Y., Murai S., Sonoda N. (1985). Synthesis utilizing reducing ability of carbon selenocarboxamides: Reaction of nitriles with selenium, carbon monoxide, and water. J. Org. Chem..

[22] Geisler K., Jacobs A., Kunzler A., Mathes M., Girrleit H., Zimmermann B., Bulka E., Pferffer W.D., Langer P. (2002). Efficient synthesis of primary selenocarboxylic amides by reaction of nitriles with phosphorous(V) selenide. Synlett.

[23] Kamminski R., Glass R.S., Skowronska A. (2001). A convenient synthesis of selenocarboxamides from nitriles. Synthesis.

[24] Cohen V.J. (1978). Synthesis of unsubstituted aromatic and heterocyclic selenocarboxamides. Synthesis.

[25] Shimada K., Hikage S., Takeishi Y., Takigawa Y. (1990). A Novel synthesis of primary selenoamides from nitriles by the treatment of bis(trimethylsilyl) selenide and BF_3_·OEt_2_. Chem. Lett..

[26] Ishihara H., Yosimuura K., Kouketsu M. (1998). A facile preparation of aliphatic and aromatic primary selenoamides using 4-methylselenobenzoate as a new selenating reagent. Chem. Lett..

[27] Gray I.P., Bhattacharyya P., Slawin A.M.Z., Woollins J.D. (2005). A new synthesis of (PhPSe_2_)_2_ (Woollis reagent) and its use in the synthesis of novel P-Se heterocycles. Chem. Eur. J..

[28] Hua G., Woollins J.D. (2009). Formation and reactivity of phosphorus-selenium rings. Angew. Chem. Int. Ed..

[29] Gomez C.J.A., Romano R.M., Beckers H., Willner H., Della V.C.O. (2010). Trifluoroselenoacetic acid, CF_3_C(O)SeH: Preparation and properties. Inorg. Chem..

[30] Abdo M., Zhang Y., Schramm V.L. (2010). Electrophilic aromatic selenylation: New OPRT inhibitors. Org. Lett..

[31] Wong R.C.S., Ooi M.L. (2011). A new approach to coordination chemistry involving phosphorus-selenium based ligands. Ring opening, deselenation and phosphorus–phosphorus coupling of Woollins’ reagen. Inorg. Chim. Acta.

[32] Hua G., Griffin J.M., Ashbrook S.E., Slawin A.M.Z., Woollins J.D. (2011). Octaselenocyclododecane. Angew. Chem. Int. Ed..

[33] Hua G., Du J., Slawin A.M.Z., Woollins J.D. (2013). Fluorinated phosphorus-selenium heteroatom compounds: Phenylphosphonofluorodiselenoic salts, adducts, and esters. Inorg. Chem..

[34] Hua G., Randall R.A.M., Slawin A.M.Z., Cordes D.B., Crawford L., Bühl M., Woollins J.D. (2013). An efficient route for the synthesis of phosphorus-selenium macroheterocycles. Chem. Commun..

[35] Hua G., Du J., Slawin A.M.Z., Woollins J.D. (2016). One-pot approach to organo-phosphorus-chalcogen macrocycles incorporating double OP(S)SC*_n_* or OP(Se)SeC*_n_* scoffolds: A synthetic and structural study. Chem. Eur. J..

[36] Hua G., Li Y., Slawin A.M.Z., Woollins J.D. (2006). Synthesis of primary arylselenoamides by reaction of aryl nitriles with Woollins’ reagent. Org. Lett..

[37] Axelle R.C., Sylvie D., Celine P., David L.G., Jean-Luc B., Roger A., Marie-Agnes S., Dennis S., Daniel M. (2002). *N*-Aryl *N′*-hydroxyguanidines, a new class of NO-donors after selective oxidation by nitric synthases: Structure-activity relationship. J. Med. Chem..

[38] Hiroyo K., Masako I., Masahiro S., Keiro H., Keiko Y., Hiroko S., Tatsuhiro T., Tsutomu I. (2002). Chemistry of *N*-hydroxyguanidines: Photo-sensitized oxygenation and reaction with nitric oxide. Helv. Chim. Acta.

[39] Garmaise D.L., Uchiyama A. (1961). Some stable dimers of substituted benzylcyanamides. Can. J. Chem..

[40] Bi X., Lopez C., Bacchi C.J., Rattendi D., Woster P.M. (2006). Novel alkylpolyaminoguanidines and alkylpolyaminobiguanides with potent antitrypanosomal activity. Bioorg. Med. Chem. Lett..

[41] Bakunov S.A., Rukavishnikov A.V., Kachev A.V. (2000). Modification of the Tieman rearrangement: One-pot synthesis of *N*,*N*-disubstituted cyanamides from amidoximes. Synthesis.

[42] Hua G., Zhang Q., Li Y., Slawin A.M.Z., Woollins J.D. (2009). Novel heterocyclic selenazadiphospholaminediselenides, zwitterionic carbamidoyl(phenyl)-phosphinodiselenoic acids and selenoureas derived from cyanamides. Tetrahedron.

[43] Kurita E., Matsuura H., Ohno K. (2004). Relationship between force constants and bond lengths for CX (X = C, Si, Ge, N, P, As, O, S, Se, F, Cl and Br) single and multiple bonds: Formulation of Badger’s rule for universal use. Spectrochim. Acta A.

[44] Koketsu M., Kanoh K., Ando H., Ishihara H. (2006). A facile synthesis of 2-amino-1,3-selenazole by reaction of *N*,*N*-unsubstituted selenourea with ketone. Heteroat. Chem..

[45] Hua G., Du J., Slawin A.M.Z., Woollins J.D. (2014). 2,4-Diaryl-1,3-chalcogen azoles bearing pentafluorosulfanyl SF5 groups: A synthetic and structural study. J. Org. Chem..

[46] Hua G., Du J., Slawin A.M.Z., Woollins J.D. (2014). A synthetic and structural study of arylselenoamides and 2,4-diaryl-1,3-selenazoles. Synlett.

[47] Geisler K., Pfeiffer W.D., Künzler A., Below H., Bulka E., Langer P. (2004). Synthesis of 1,3-selenazoles and bis(selenazoles) from primary selenocarboxylic amides and selenourea. Synthesis.

[48] Murai T., Yamaguchi K., Hori F., Maruyama T. (2014). Reaction of selenoamide dianions with thio- and selenoformamides leading to the formation of 5-aminoselenazoles: Photophysical and electrochemical properties. J. Org. Chem..

[49] Wirth T. (2012). Organoselenium Chemistry.

[50] Koketsu M., Mio T., Ishihara H. (2004). Facile preparation of 1,3-selenazole-5-carboxylic acids and the carboxylates by reaction of selenazadienes with chloroacetyl chloride. Synthesis.

[51] Li G.M., Zingaro R.A., Segi M., Reibenspies J.H., Nakajima T. (1997). Synthesis and structure of telluroamides and selenoamides. The first crystallographic study of Telluroamides. Organometallics.

[52] Sheldrick G.M. (2015). Crystal structure refinement with *SHELXL*. Acta Crystallogr. Sect. C.

