# Supplementary material for: Synthesis and Single Crystal Structures of Substituted-1,3-Selenazol-2-amines
Source: Molecules. 2016 Dec 29;22(1):46. doi: 10.3390/molecules22010046 (PMC6155636; doi:10.3390/molecules22010046)
Supplement: Supplementary file 1 [file molecules-22-00046-s001.pdf]

Supplementary Materials: Synthesis and Single Crystal Structures of Substituted-1,3-Selenazol-2-amines

Guoxiong Hua, Junyi Du, Alexandra M. Z. Slawin and J. Derek Woollins

^{1}H-, ^{13}C-NMR Spectra of Compounds 5–16.

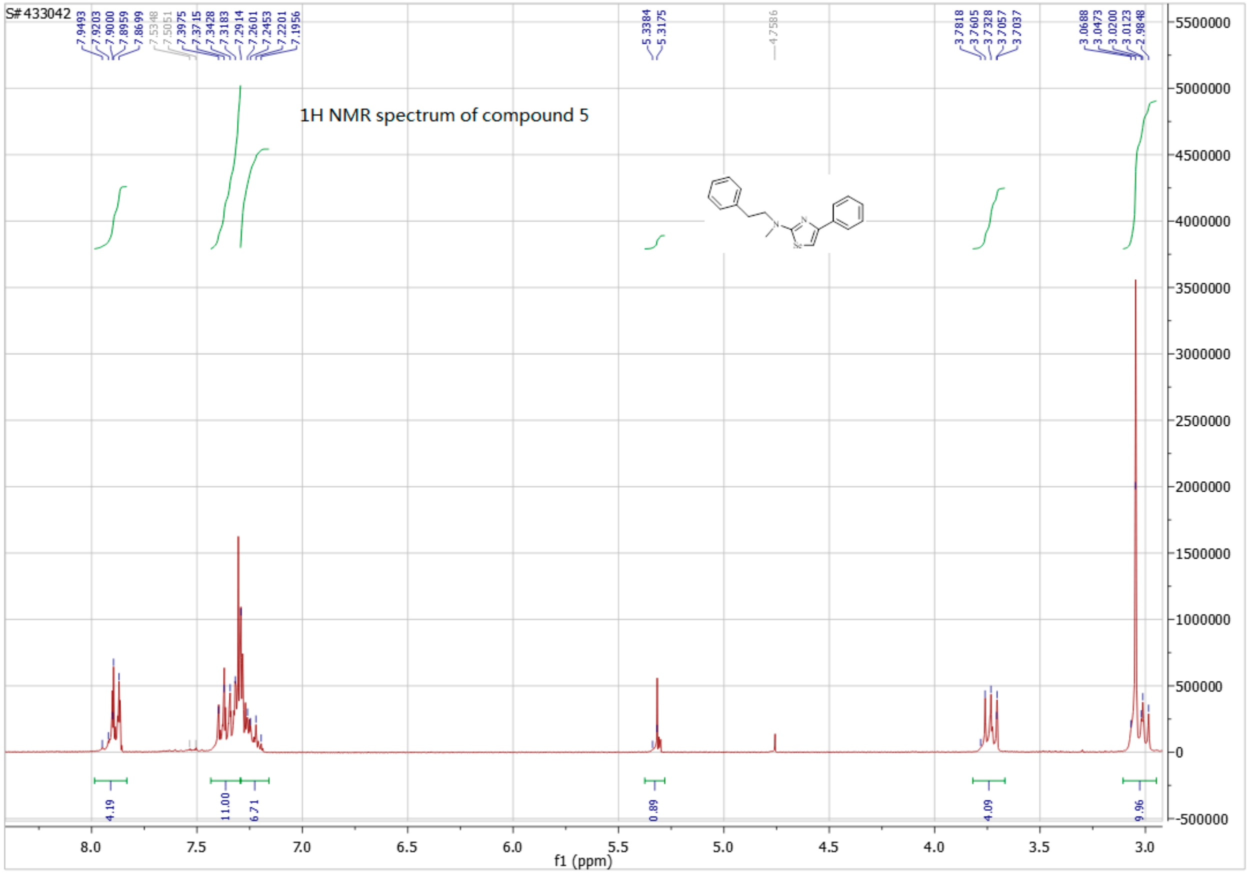

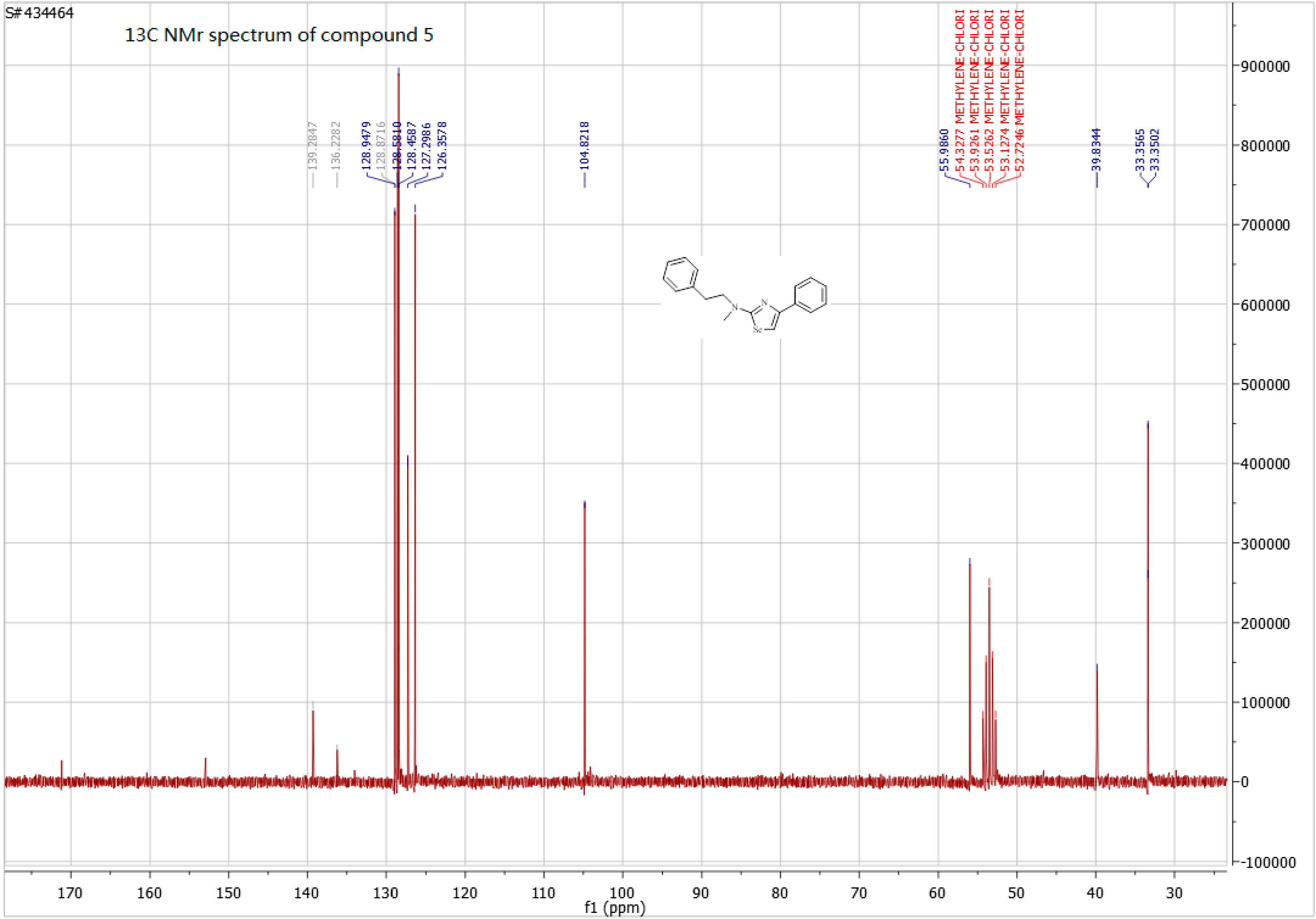
S#434464
13C NMr spectrum of compound 5
139.2847
136.2282
128.9479
128.8716
128.5810
128.4587
127.2986
126.3578
104.8218
55.9860
54.3277 METHYLENE-CHLORI
53.9261 METHYLENE-CHLORI
53.5262 METHYLENE-CHLORI
53.1274 METHYLENE-CHLORI
52.7246 METHYLENE-CHLORI
39.8344
33.3565
33.3502
900000
800000
700000
600000
500000
400000
300000
200000
100000
0
−100000
170
160
150
140
130
120
110
100
90
80
70
60
50
40
30
f1 (ppm)

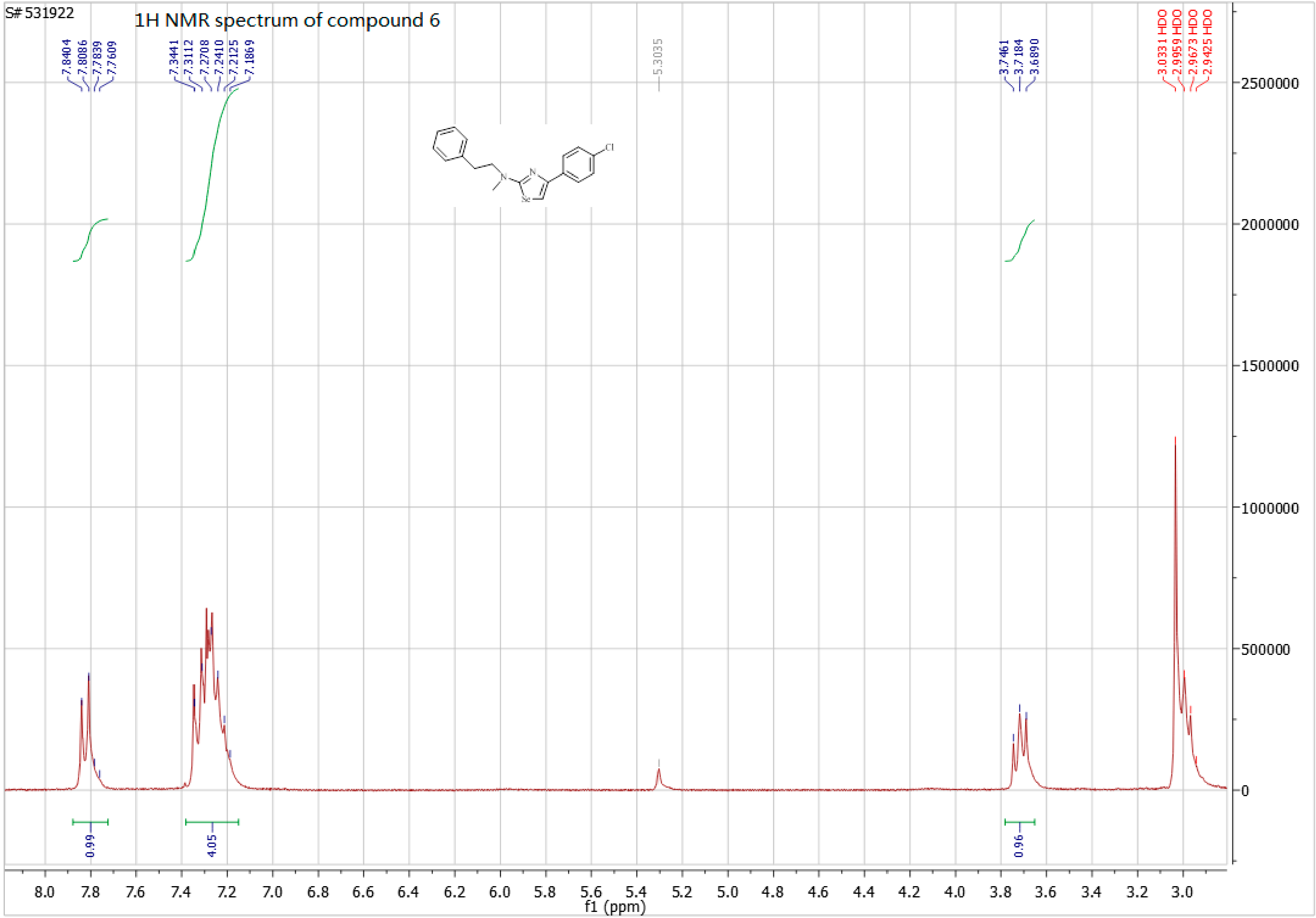
S# 531922
1H NMR spectrum of compound 6
7.8404
7.8086
7.7839
7.7609
7.3441
7.3112
7.2708
7.2410
7.2125
7.1869
5.3035
3.7461
3.7184
3.6890
3.0331 HDO
2.9959 HDO
2.9673 HDO
2.9425 HDO
2500000
2000000
1500000
1000000
500000
0
0.99
4.05
0.96
8.0 7.8 7.6 7.4 7.2 7.0 6.8 6.6 6.4 6.2 6.0 5.8 5.6 5.4 5.2 5.0 4.8 4.6 4.4 4.2 4.0 3.8 3.6 3.4 3.2 3.0
f1 (ppm)

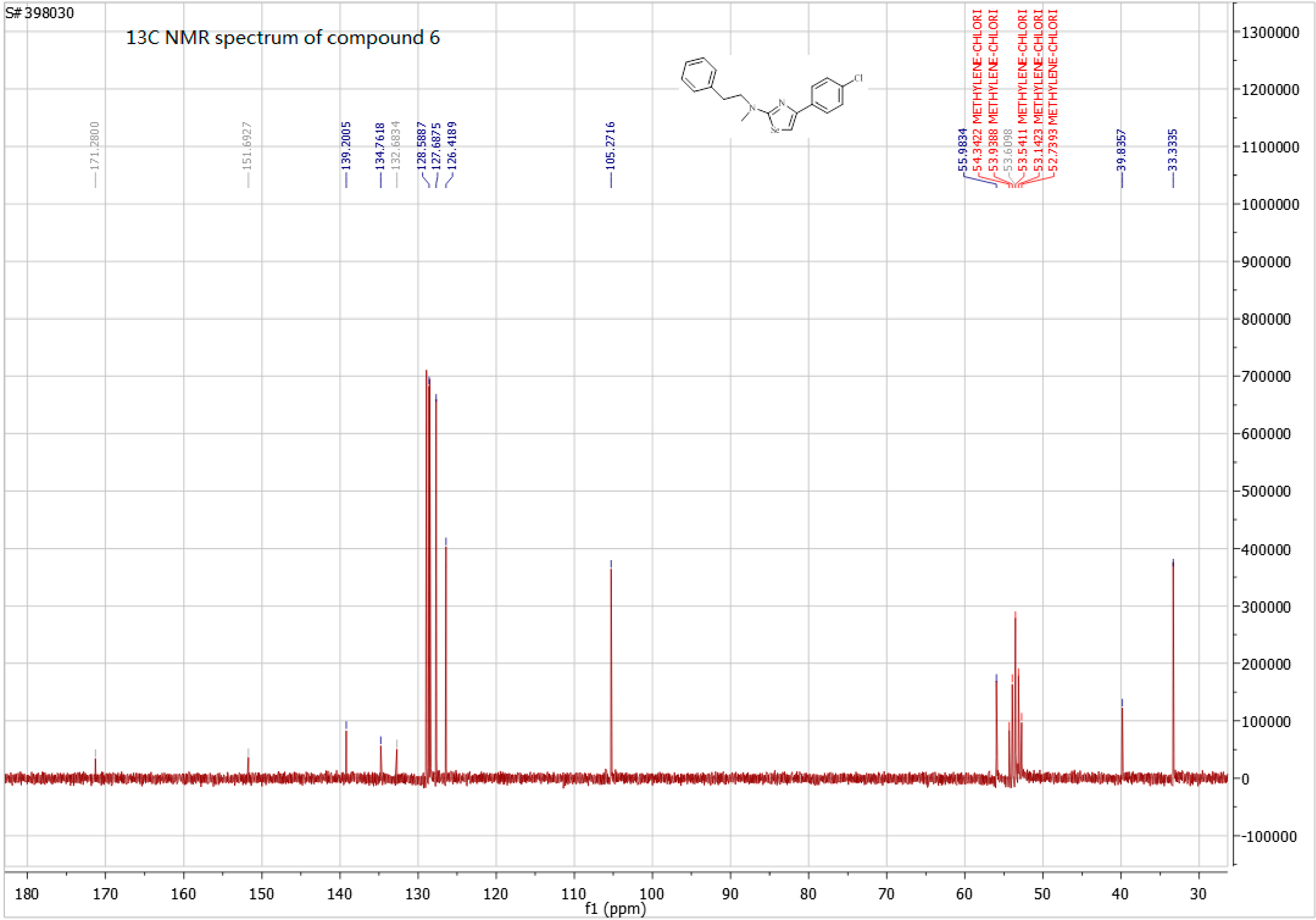
S# 398030
13C NMR spectrum of compound 6
171.2800
151.6927
139.2005
134.7618
132.6834
128.5887
127.6875
126.4189
105.2716
55.9834
54.3422 METHYLENE-CHLORI
53.9388 METHYLENE-CHLORI
53.6098
53.5411 METHYLENE-CHLORI
53.1423 METHYLENE-CHLORI
52.7393 METHYLENE-CHLORI
39.8357
33.3335
f1 (ppm)

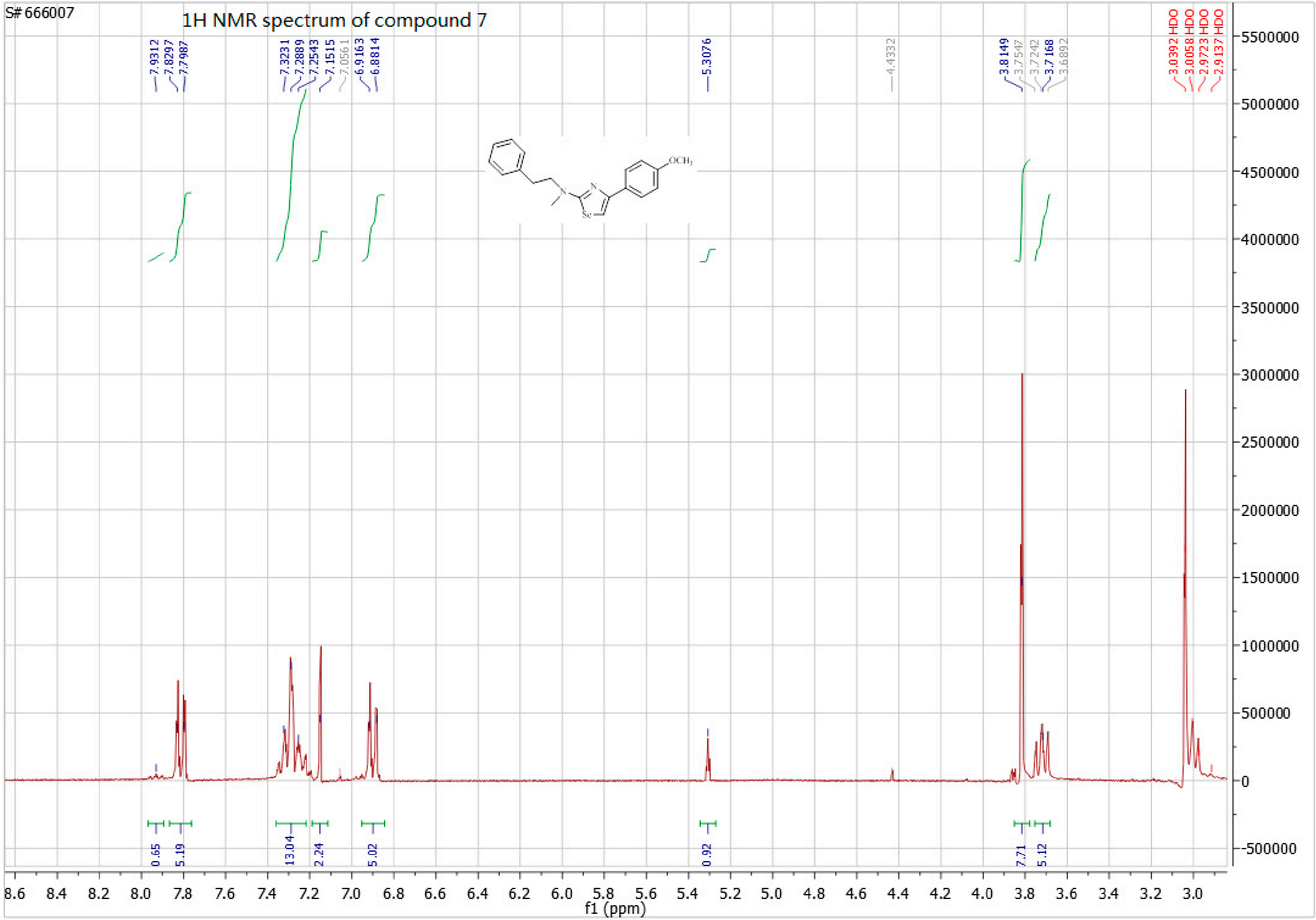
S# 666007
1H NMR spectrum of compound 7
OCH3
Se
N
f1 (ppm)

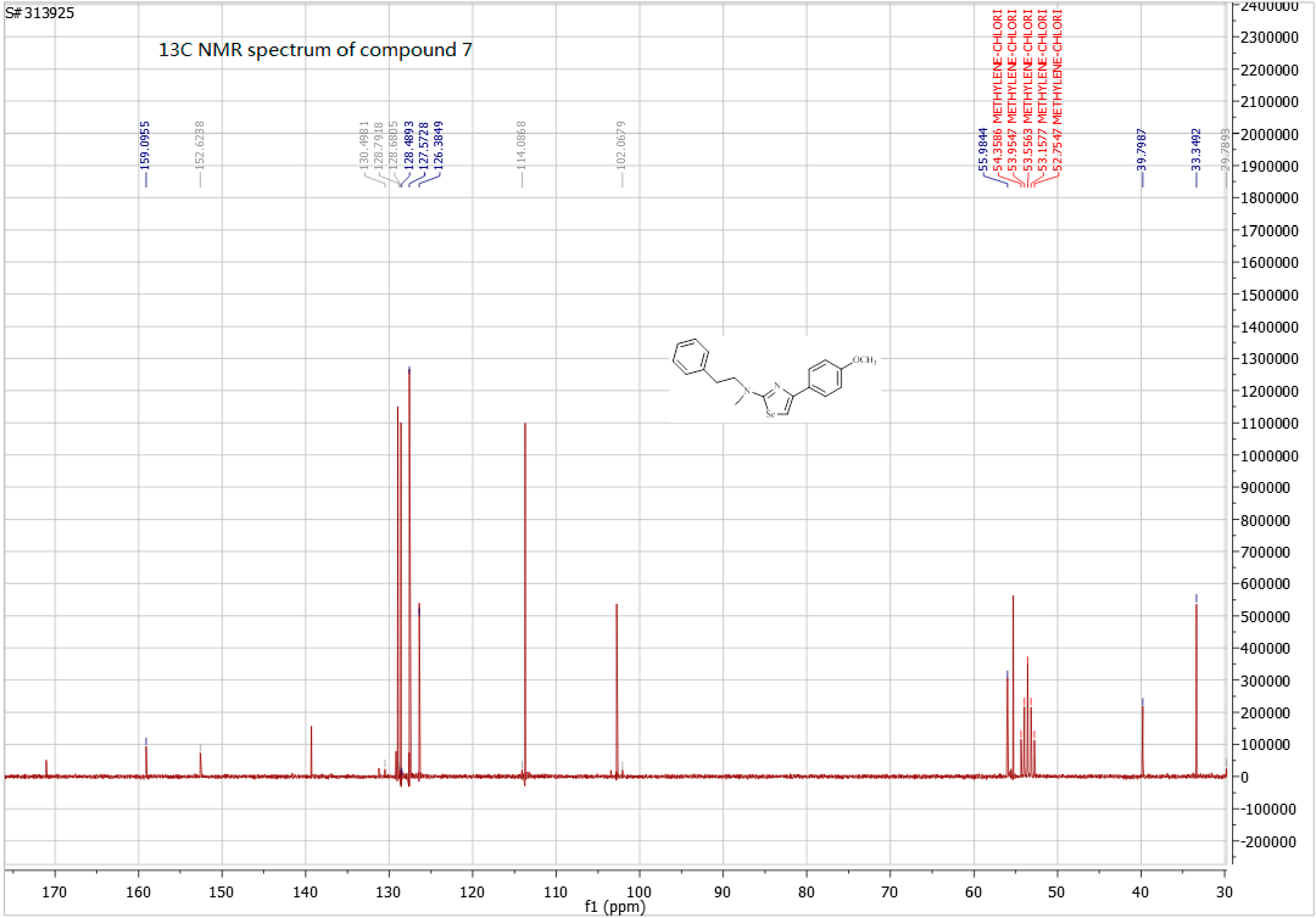
S# 313925
13C NMR spectrum of compound 7
159.0955
152.6238
130.4981
128.7918
128.6805
128.4893
127.5728
126.3849
114.0868
102.0679
55.9844
54.3586 METHYLENE-CHLORI
53.9547 METHYLENE-CHLORI
53.5563 METHYLENE-CHLORI
53.1577 METHYLENE-CHLORI
52.7547 METHYLENE-CHLORI
39.7987
33.3492
29.7893
OCH3
N
Se
f1 (ppm)

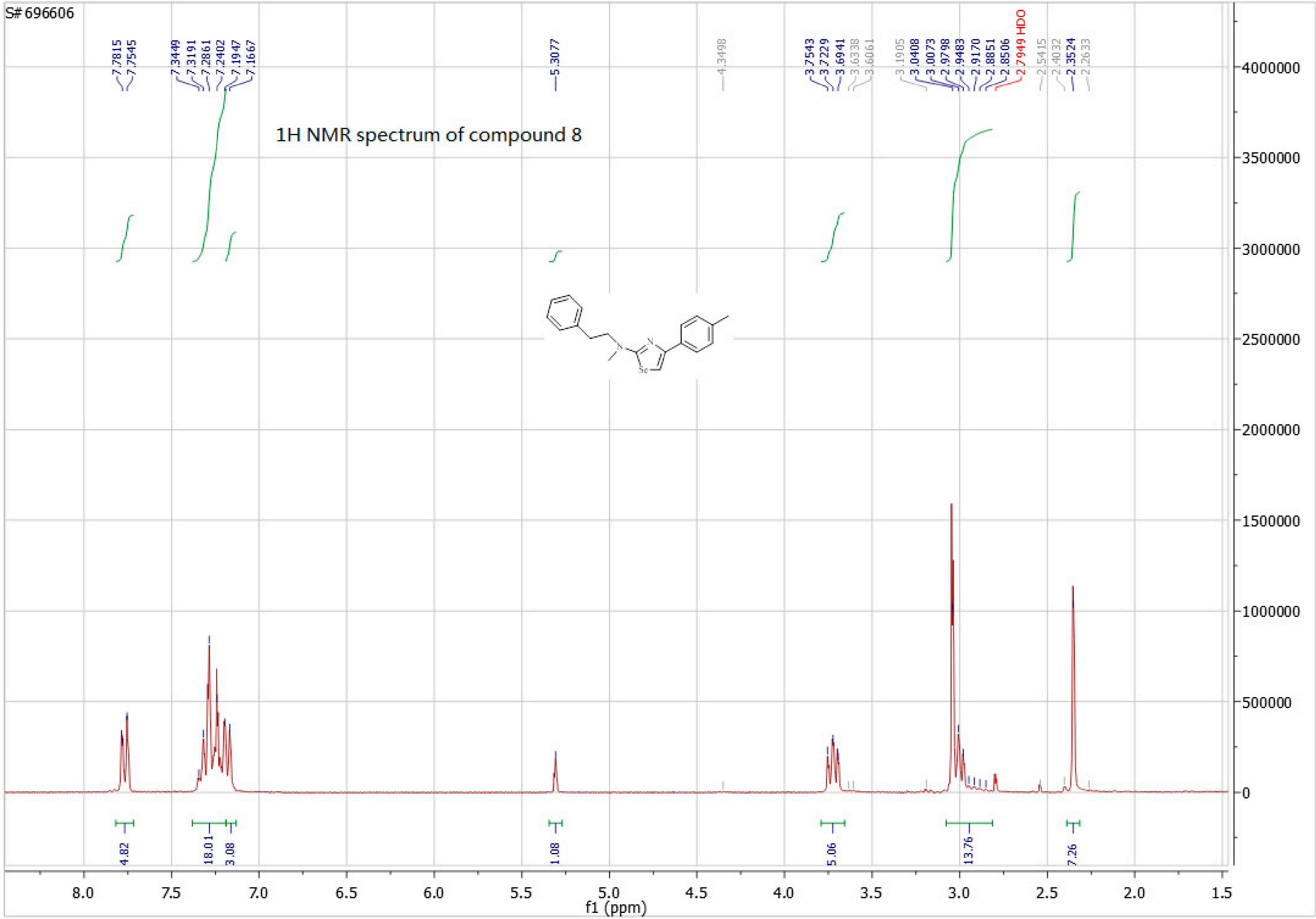
S# 696606
1H NMR spectrum of compound 8
7.7815
7.7545
7.3449
7.3191
7.2861
7.2402
7.1947
7.1667
5.3077
4.3498
3.7543
3.7229
3.6941
3.6338
3.6061
3.1905
3.0408
3.0073
2.9798
2.9483
2.9170
2.8851
2.8506
2.7949 HDO
2.5415
2.4032
2.3524
2.2633
4.82
18.01
3.08
1.08
5.06
13.76
7.26
f1 (ppm)

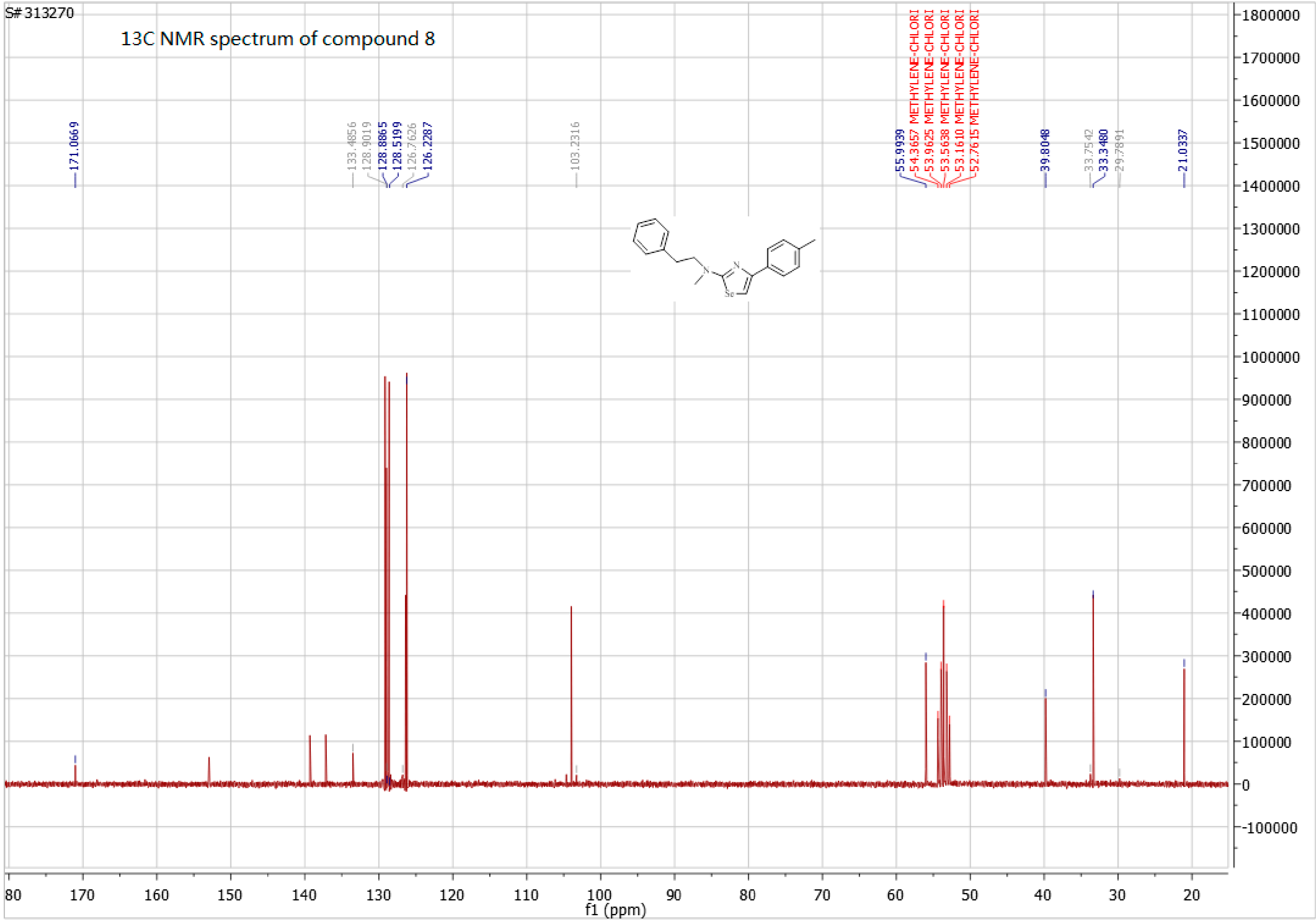
S# 313270
13C NMR spectrum of compound 8
171.0669
133.4856
128.9019
128.8865
128.5199
126.7626
126.2287
103.2316
55.9939
54.3657 METHYLENE-CHLORI
53.9625 METHYLENE-CHLORI
53.5638 METHYLENE-CHLORI
53.1610 METHYLENE-CHLORI
52.7615 METHYLENE-CHLORI
39.8048
33.7542
33.3480
29.7891
21.0337
f1 (ppm)

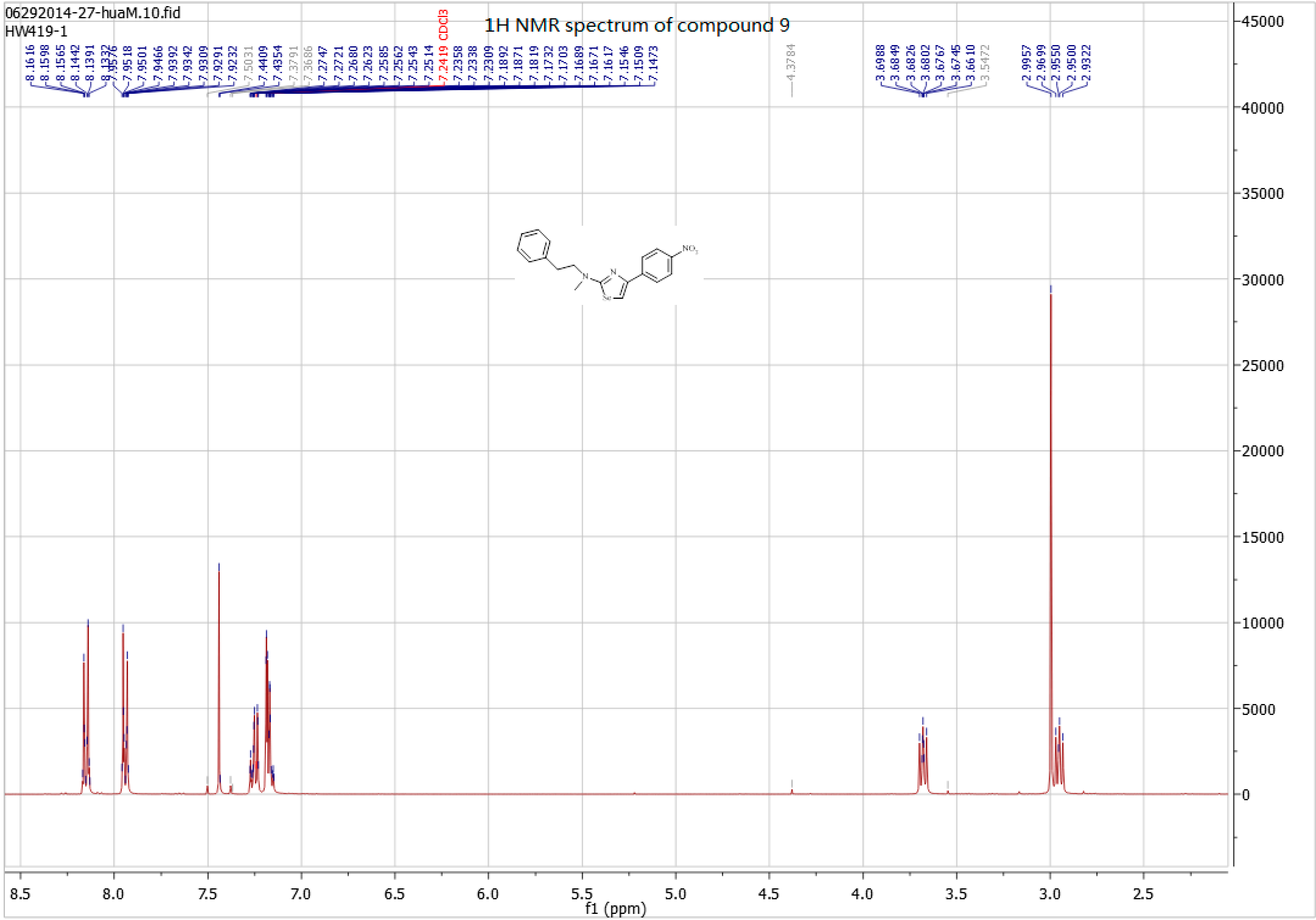
06292014-27-huaM.10.fid
HW419-1
1H NMR spectrum of compound 9
CDCl3
f1 (ppm)

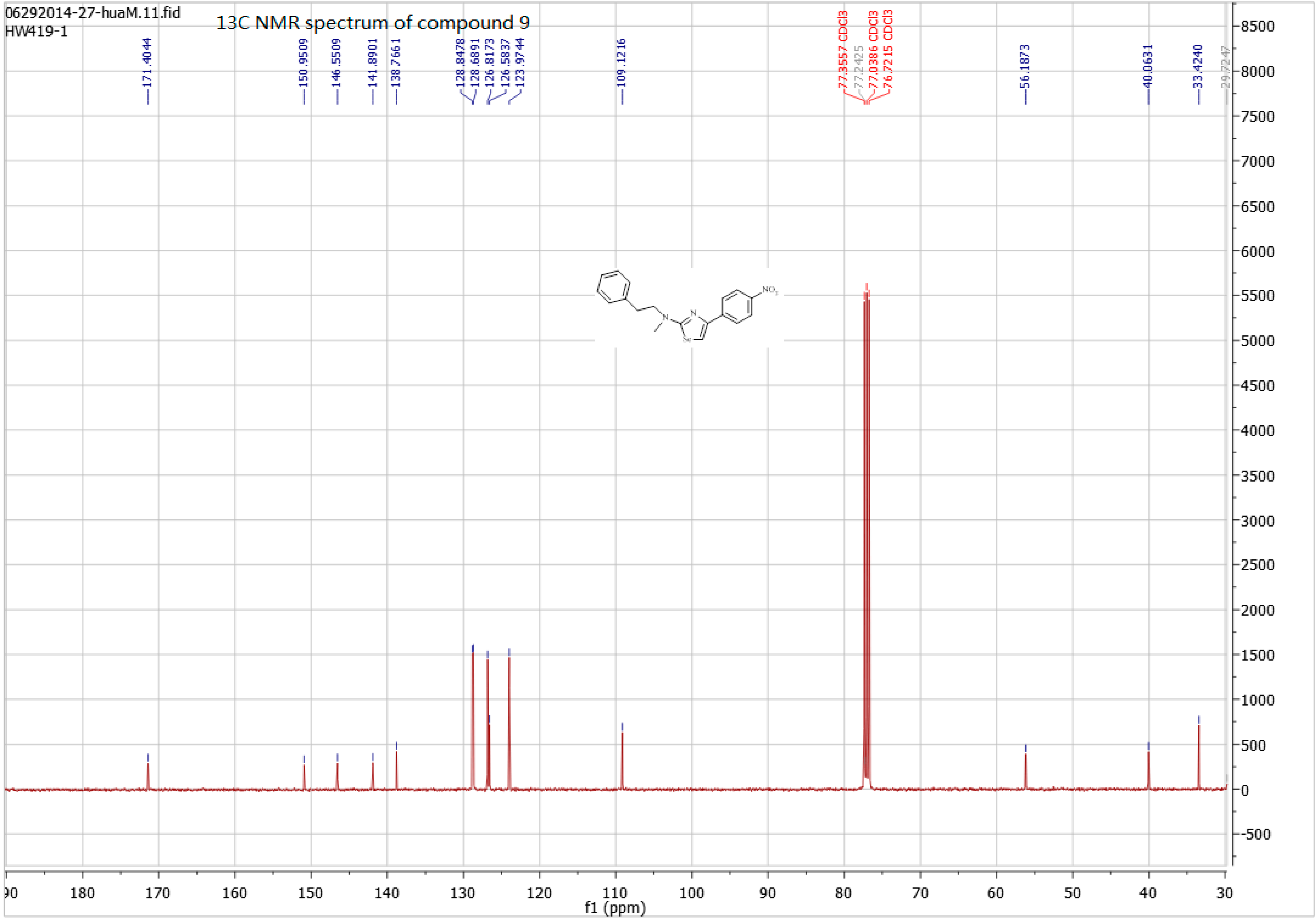

06292014-27-huaM.11.fid
HW419-1
13C NMR spectrum of compound 9
171.4044
150.9509
146.5509
141.8901
138.7661
128.8478
128.6891
126.8173
126.5837
123.9744
109.1216
77.3557 CDCl3
77.2425
77.0386 CDCl3
76.7215 CDCl3
56.1873
40.0631
33.4240
29.7247
NO2
N
f1 (ppm)

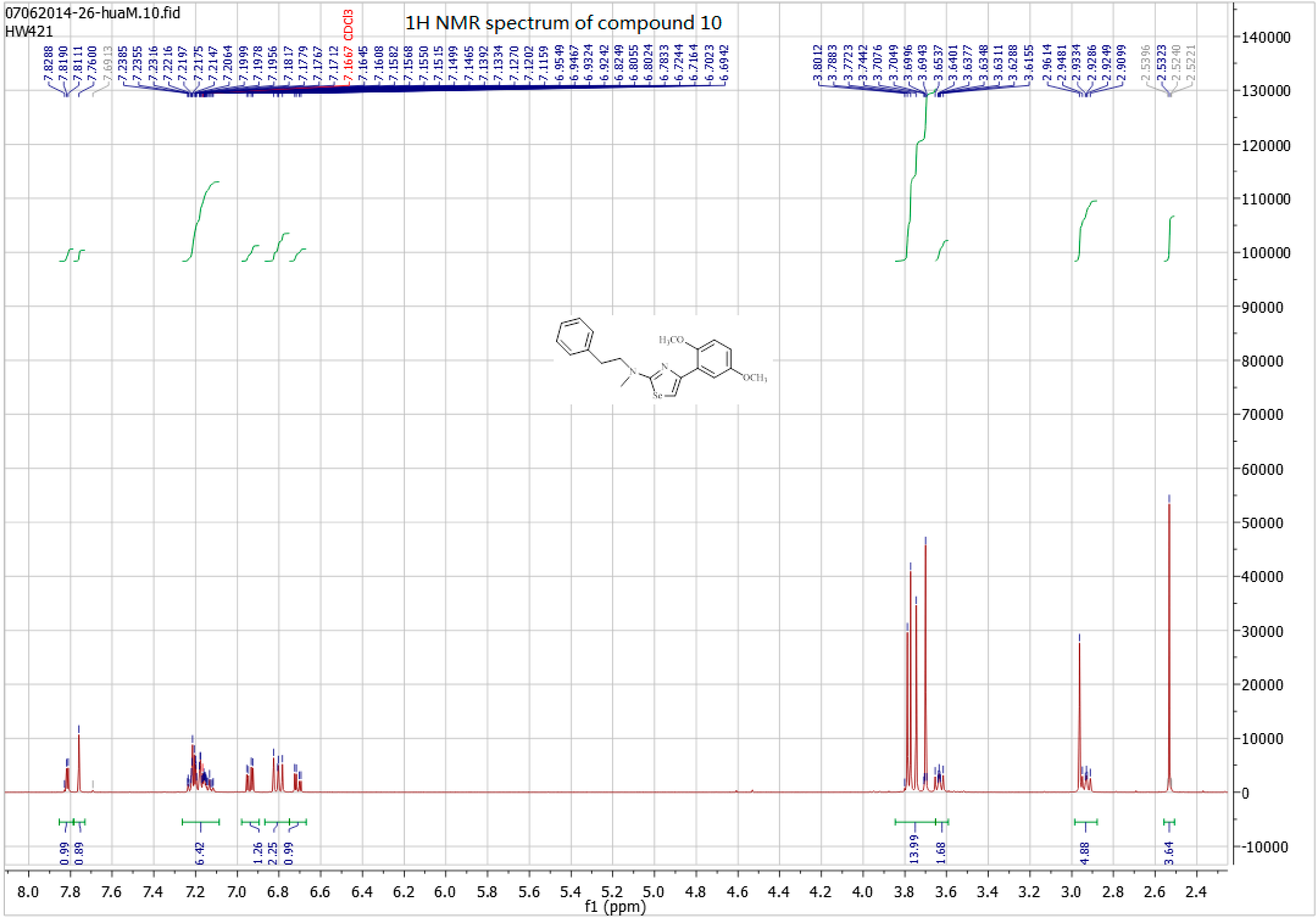
07062014-26-huaM.10.fid
HW421
1H NMR spectrum of compound 10
CDCl3
H3CO
OCH3
N
Se
f1 (ppm)

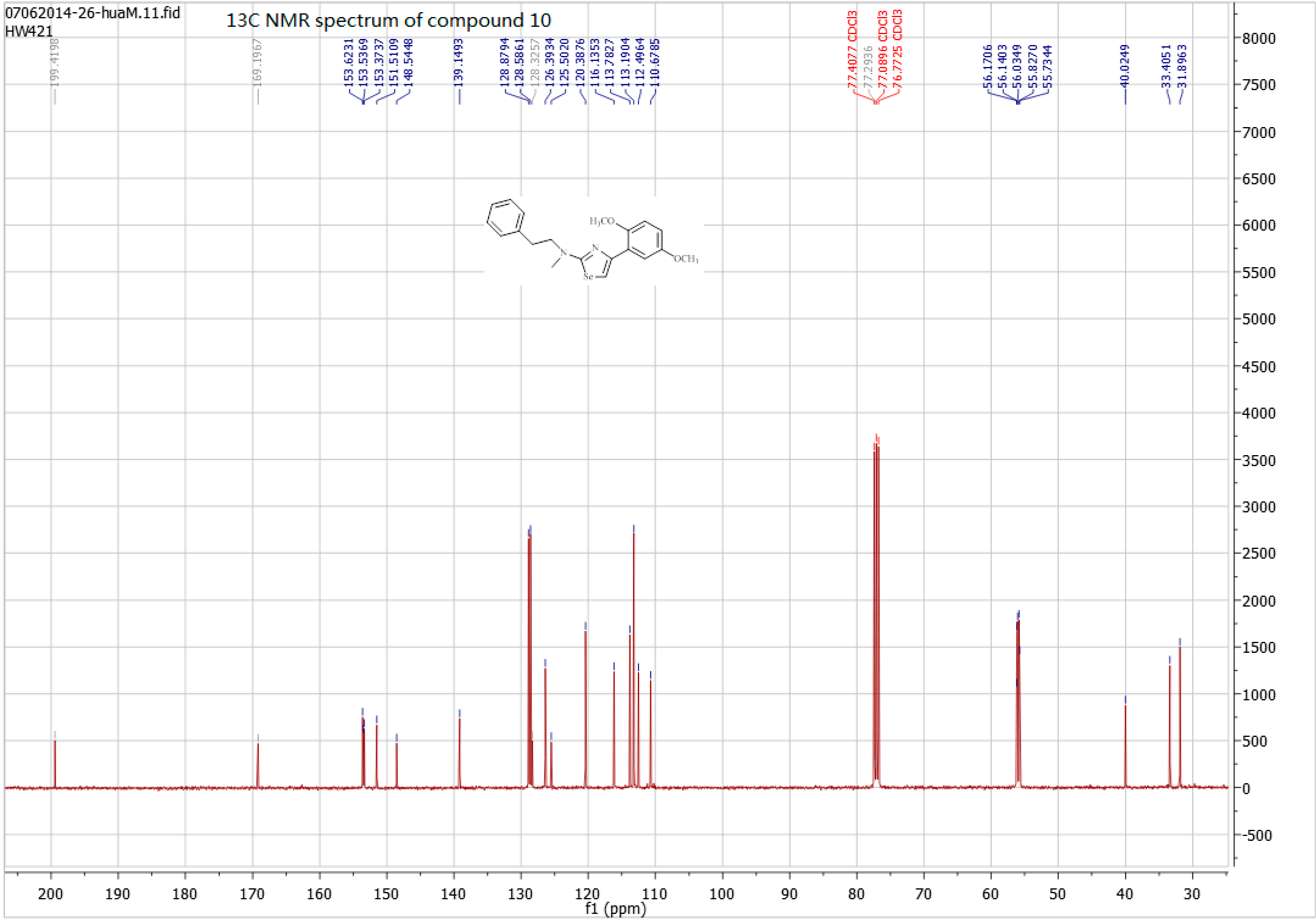
07062014-26-huaM.11.fid
HW421
13C NMR spectrum of compound 10
199.4198
169.1967
153.6231
153.5369
153.3737
151.5109
148.5448
139.1493
128.8794
128.5861
128.3257
126.3934
125.5020
120.3876
116.1353
113.7827
113.1904
112.4964
110.6785
77.4077 CDCl3
77.2936
77.0896 CDCl3
76.7725 CDCl3
56.1706
56.1403
56.0349
55.8270
55.7344
40.0249
33.4051
31.8963
f1 (ppm)

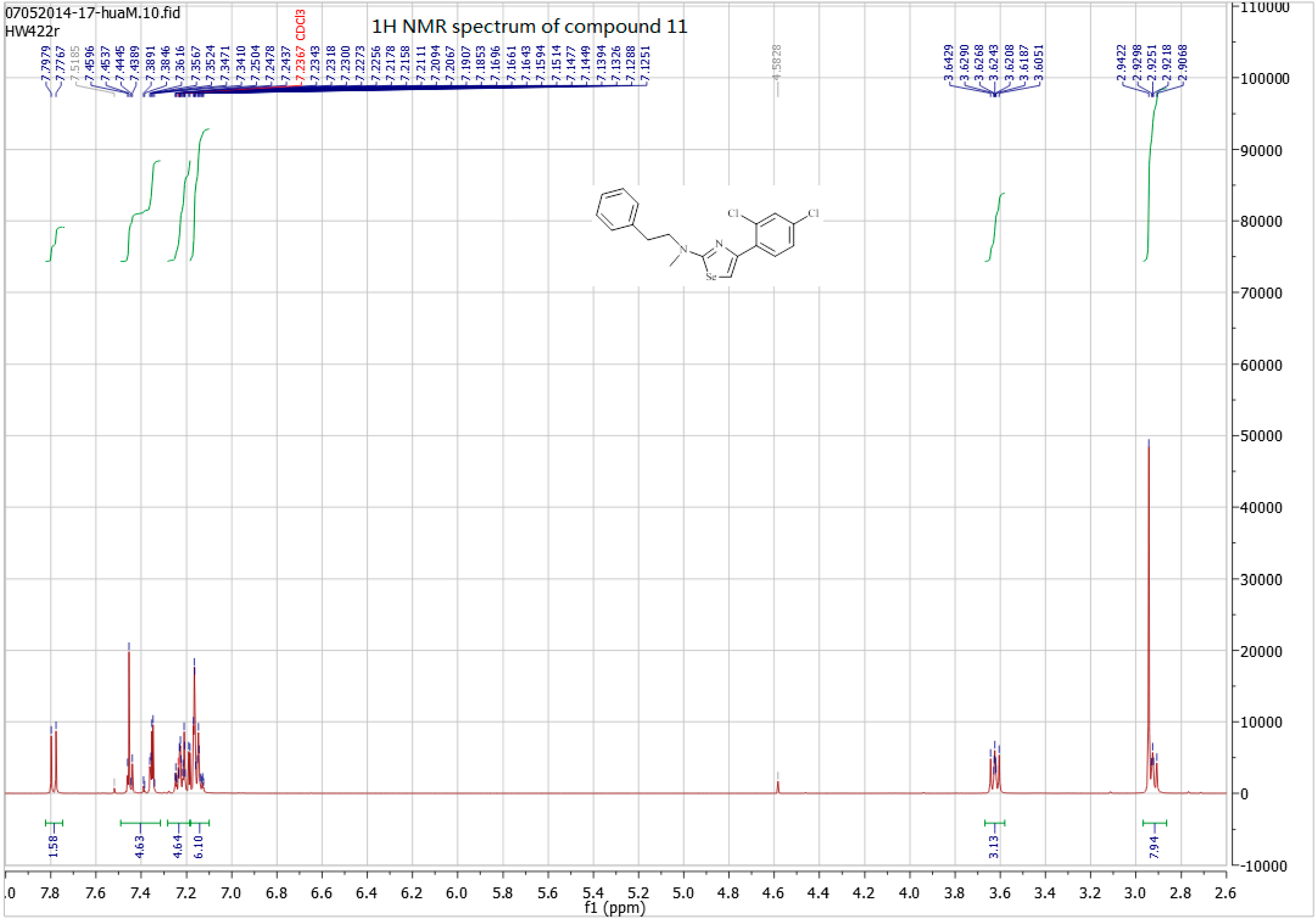

07052014-17-huaM.10.fid
HW422r
1H NMR spectrum of compound 11
CDCl3
f1 (ppm)

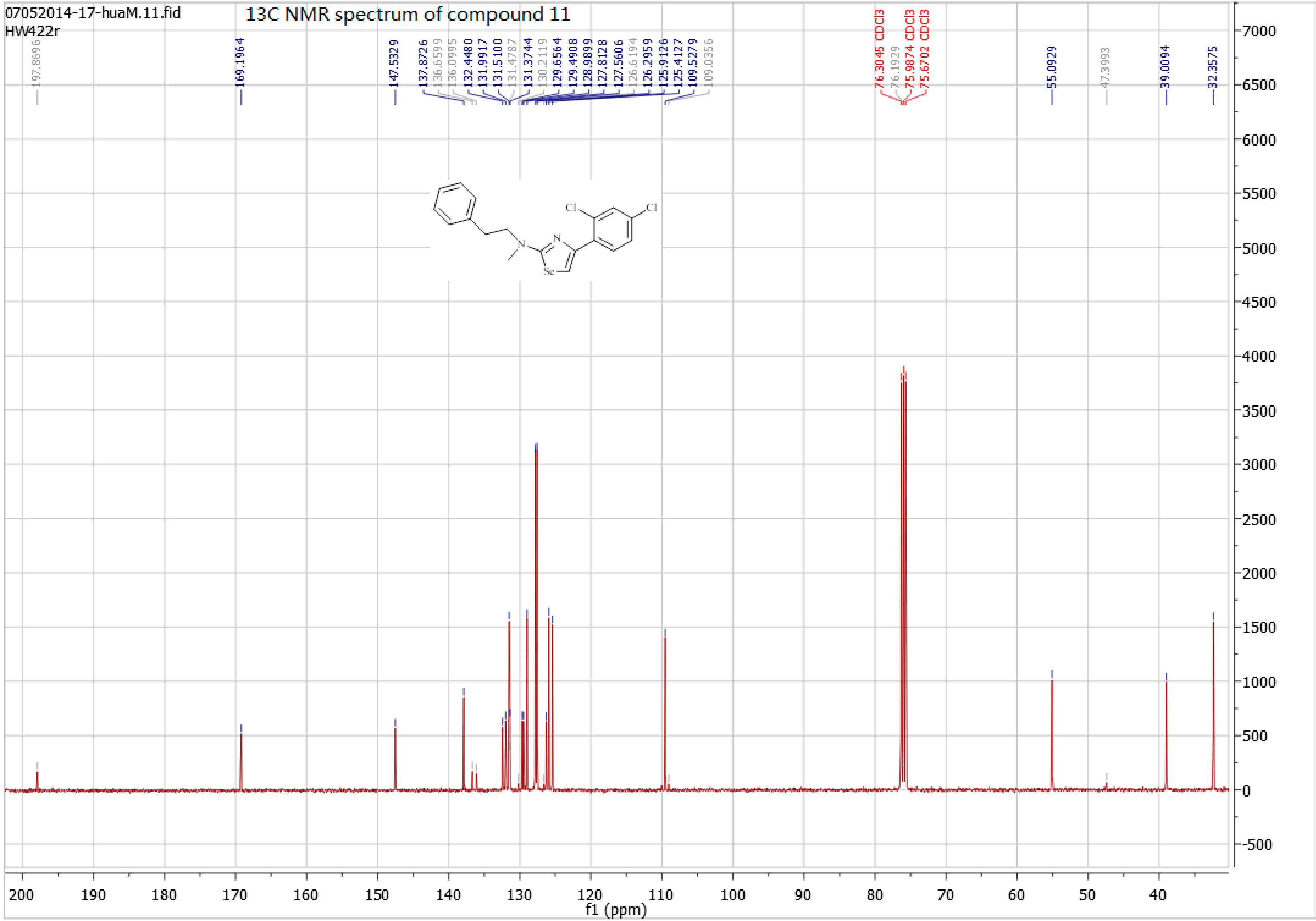

07052014-17-huaM.11.fid
HW422r
13C NMR spectrum of compound 11
f1 (ppm)

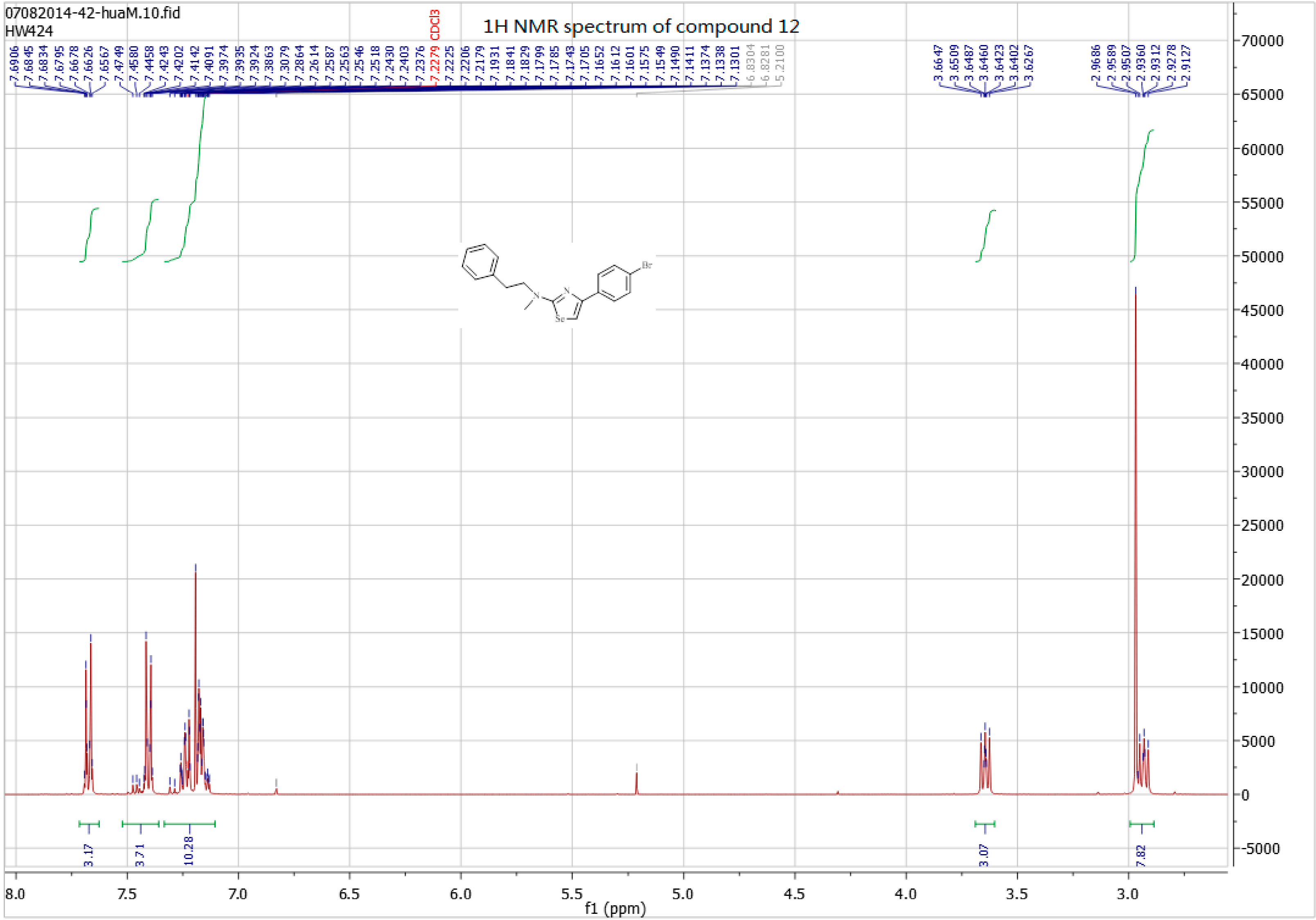
07082014-42-huaM.10.fid
HW424
1H NMR spectrum of compound 12
CDCl3
Br
N
Se
3.17
3.71
10.28
3.07
7.82
f1 (ppm)

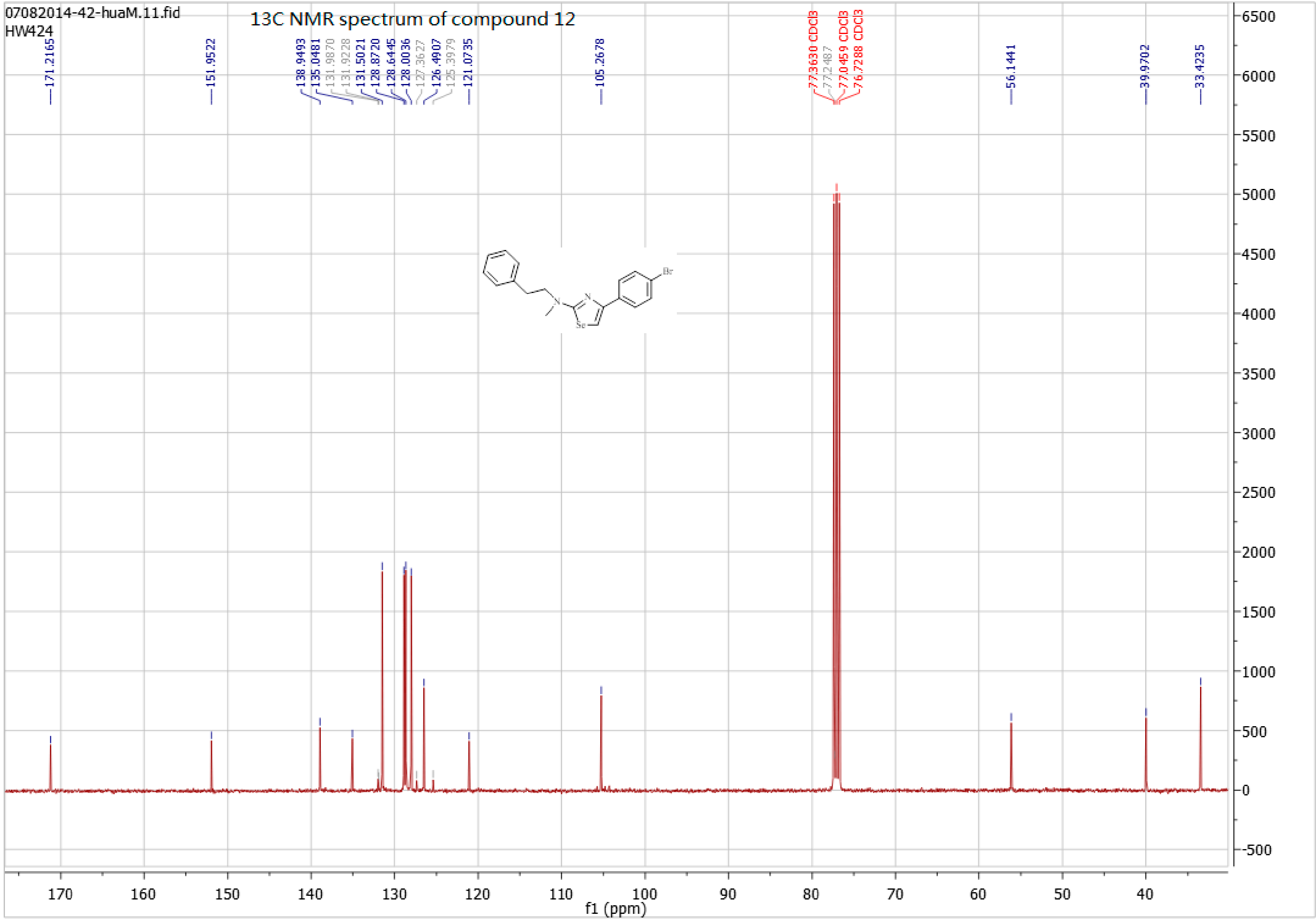

07082014-42-huaM.11.fid
HW424
13C NMR spectrum of compound 12
171.2165
151.9522
138.9493
135.0481
131.9870
131.9228
131.5021
128.8720
128.6445
128.0036
127.3627
126.4907
125.3979
121.0735
105.2678
77.3630 CDCl3
77.2487
77.0459 CDCl3
76.7288 CDCl3
56.1441
39.9702
33.4235
Br
N
Se
f1 (ppm)

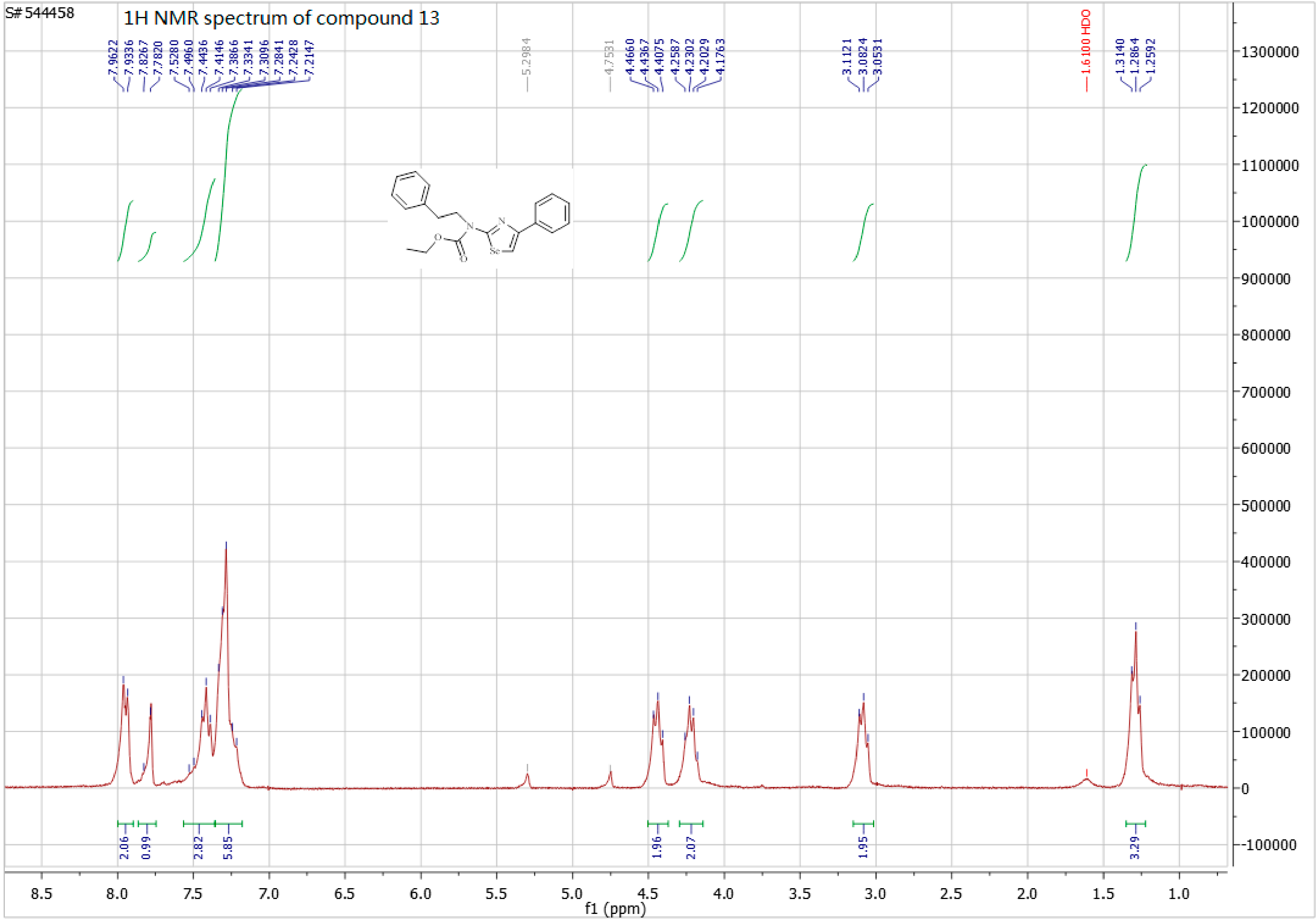
S# 544458
1H NMR spectrum of compound 13
f1 (ppm)

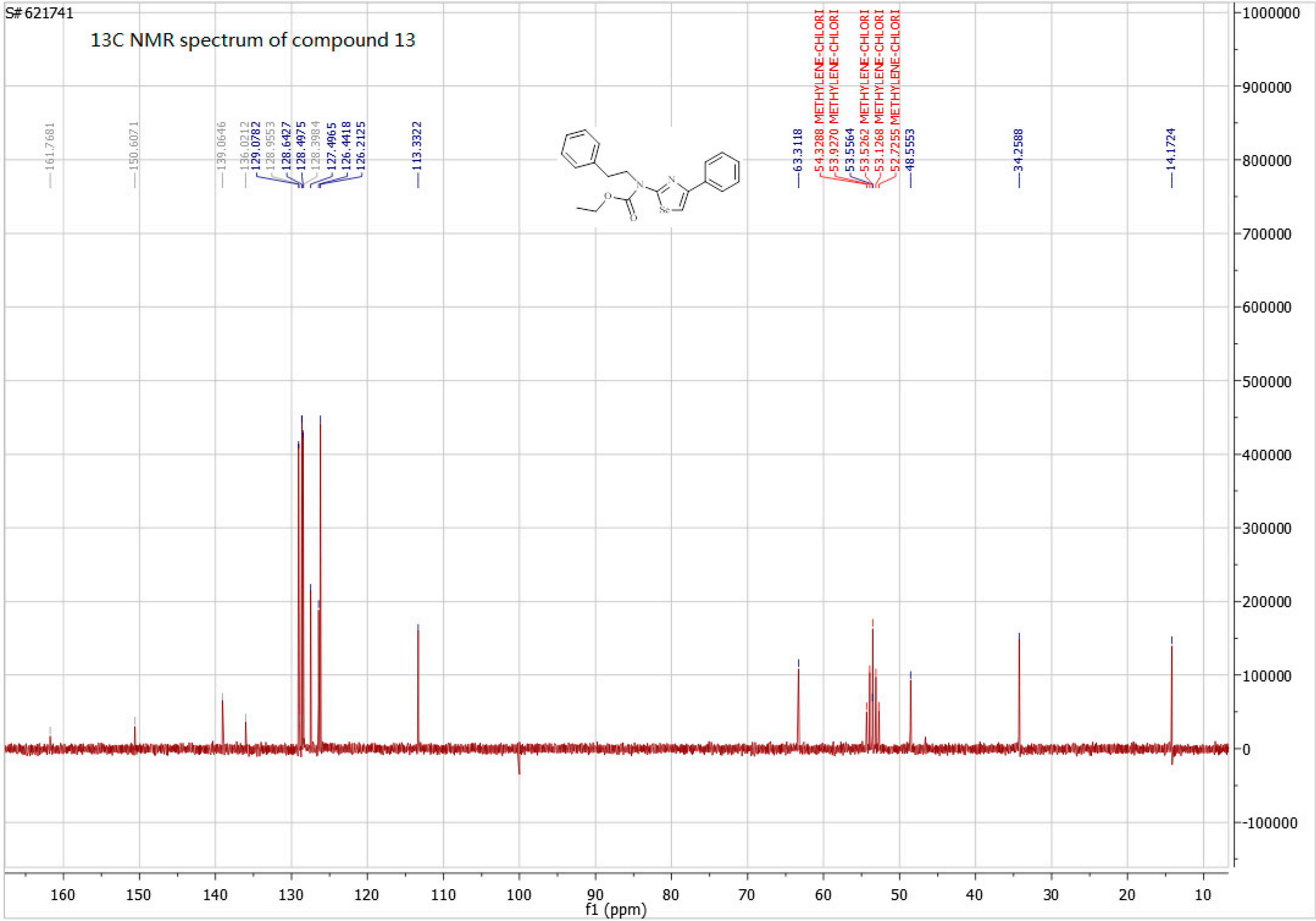
S# 621741
13C NMR spectrum of compound 13
161.7681
150.6071
139.0646
136.0212
129.0782
128.9553
128.6427
128.4975
128.3984
127.4965
126.4418
126.2125
113.3322
63.3118
54.3288 METHYLENE-CHLORI
53.9270 METHYLENE-CHLORI
53.5564
53.5262 METHYLENE-CHLORI
53.1268 METHYLENE-CHLORI
52.7255 METHYLENE-CHLORI
48.5553
34.2588
14.1724
f1 (ppm)

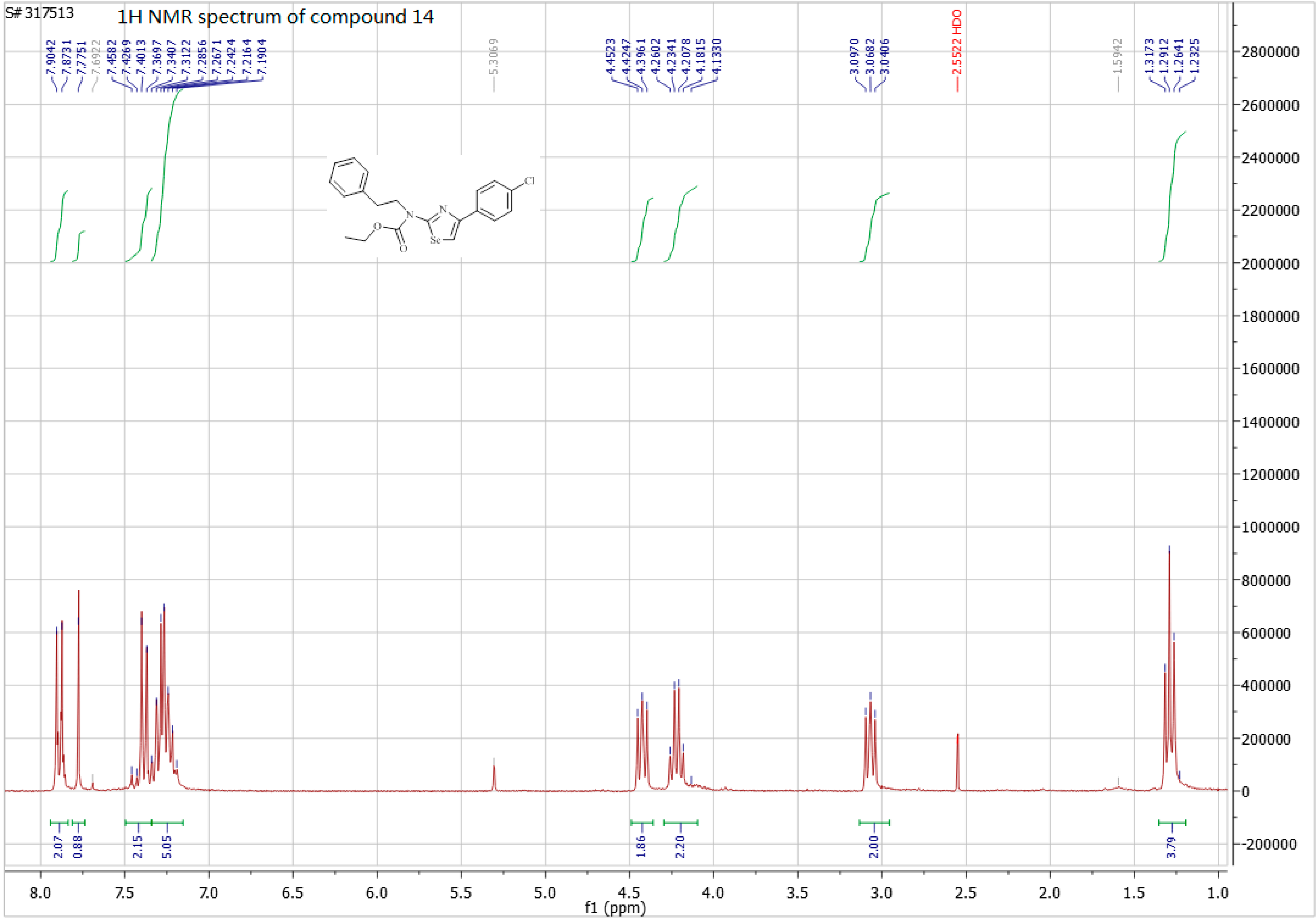

S# 317513
1H NMR spectrum of compound 14
7.9042
7.8731
7.7751
7.6922
7.4582
7.4269
7.4013
7.3697
7.3407
7.3122
7.2856
7.2671
7.2424
7.2164
7.1904
5.3069
4.4523
4.4247
4.3961
4.2602
4.2341
4.2078
4.1815
4.1330
3.0970
3.0682
3.0406
2.5522 HDO
1.5942
1.3173
1.2912
1.2641
1.2325
2.07
0.88
2.15
5.05
1.86
2.20
2.00
3.79
f1 (ppm)

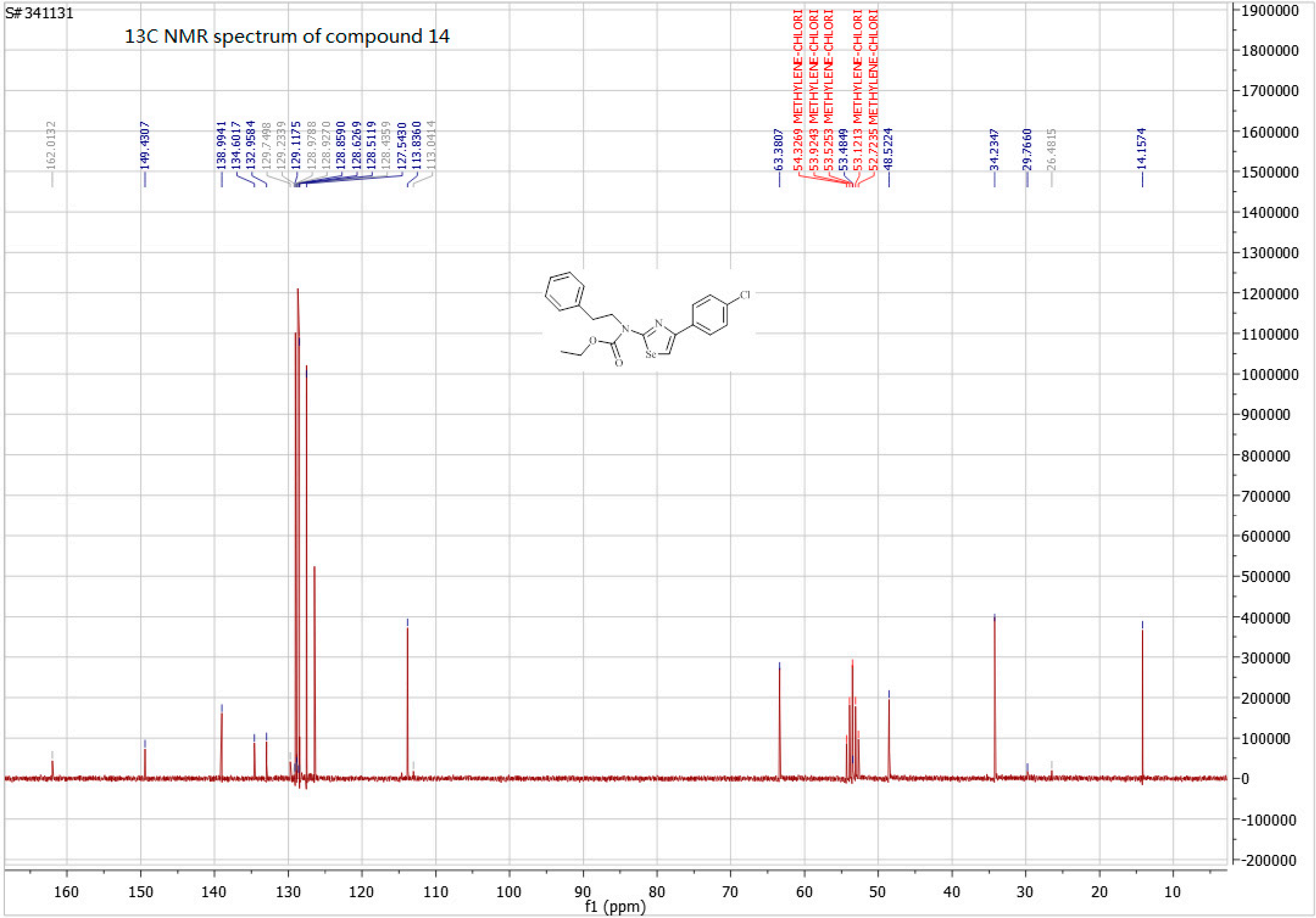

S# 341131
13C NMR spectrum of compound 14
162.0132
149.4307
138.9941
134.6017
132.9584
129.7498
129.2339
129.1175
128.9788
128.9270
128.8590
128.6269
128.5119
128.4359
127.5430
113.8360
113.0414
63.3807
54.3269 METHYLENE-CHLORI
53.9243 METHYLENE-CHLORI
53.5253 METHYLENE-CHLORI
53.4849
53.1213 METHYLENE-CHLORI
52.7235 METHYLENE-CHLORI
48.5224
34.2347
29.7660
26.4815
14.1574
f1 (ppm)

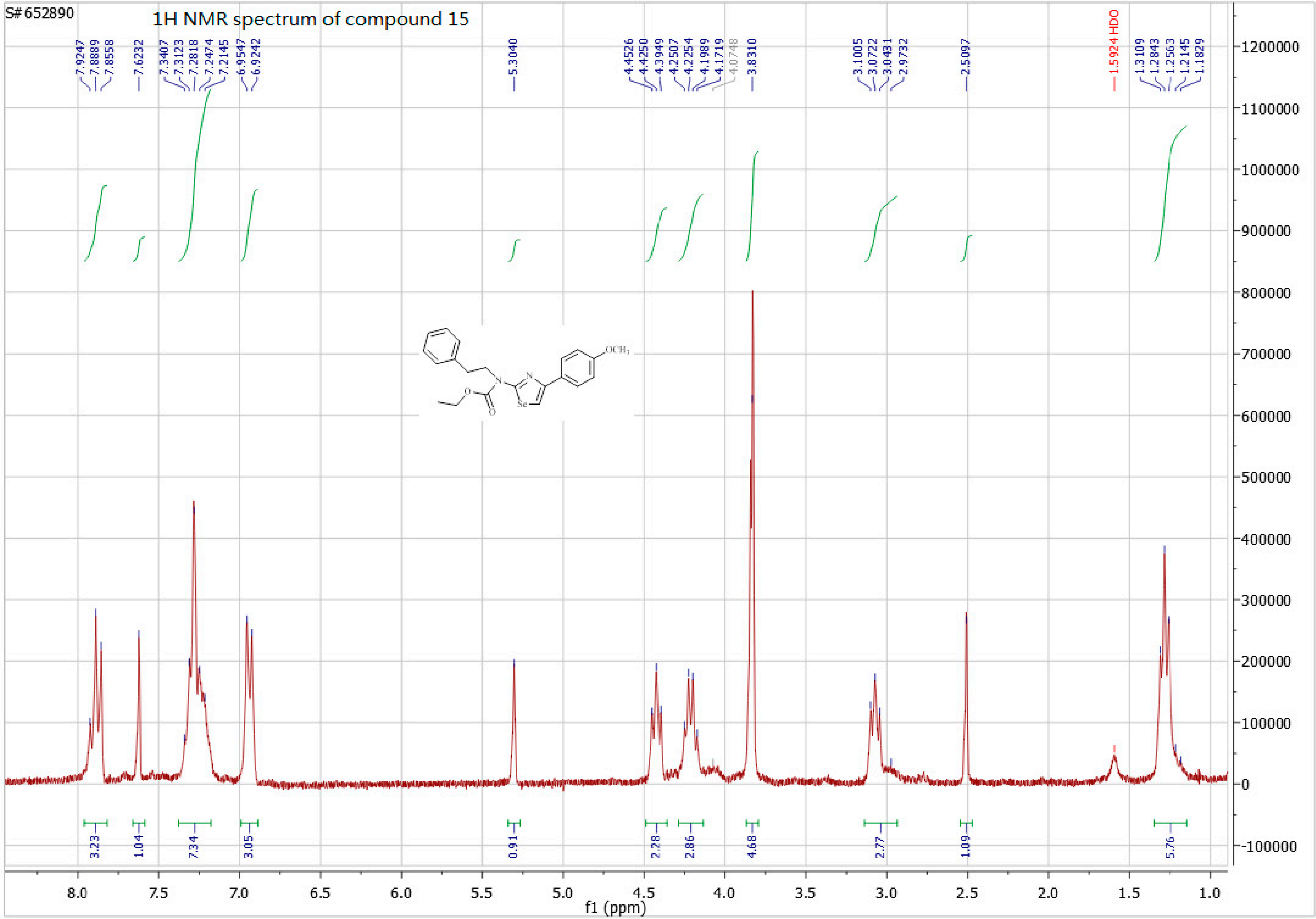
S# 652890
1H NMR spectrum of compound 15
f1 (ppm)

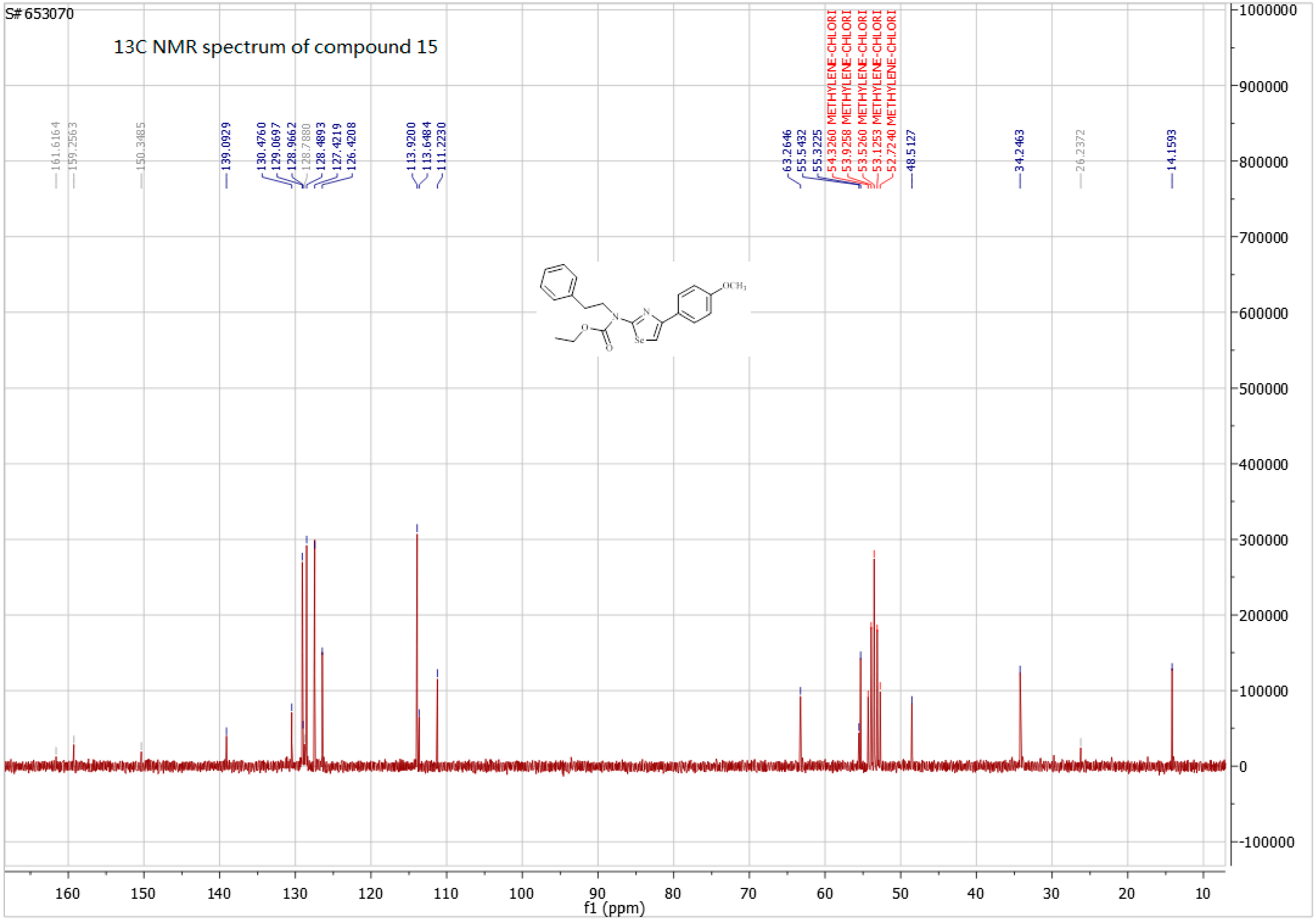
S# 653070
13C NMR spectrum of compound 15
161.6164
159.2563
150.3485
139.0929
130.4760
129.0697
128.9662
128.7880
128.4893
127.4219
126.4208
113.9200
113.6484
111.2230
63.2646
55.5432
55.3225
54.3260 METHYLENE-CHLORI
53.9258 METHYLENE-CHLORI
53.5260 METHYLENE-CHLORI
53.1253 METHYLENE-CHLORI
52.7240 METHYLENE-CHLORI
48.5127
34.2463
26.2372
14.1593
f1 (ppm)

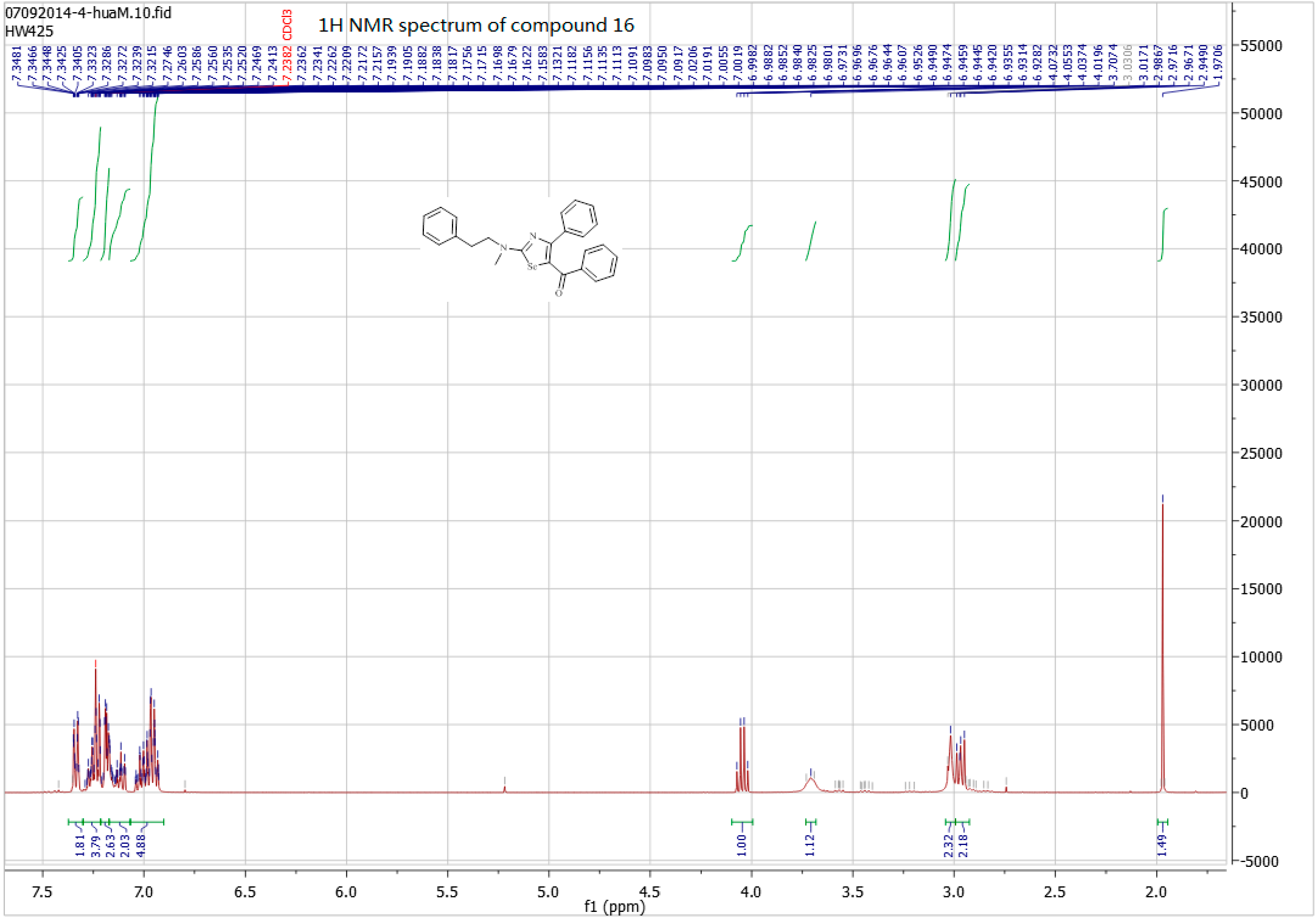
07092014-4-huaM.10.fid
HW425
1H NMR spectrum of compound 16
CDCl3
f1 (ppm)

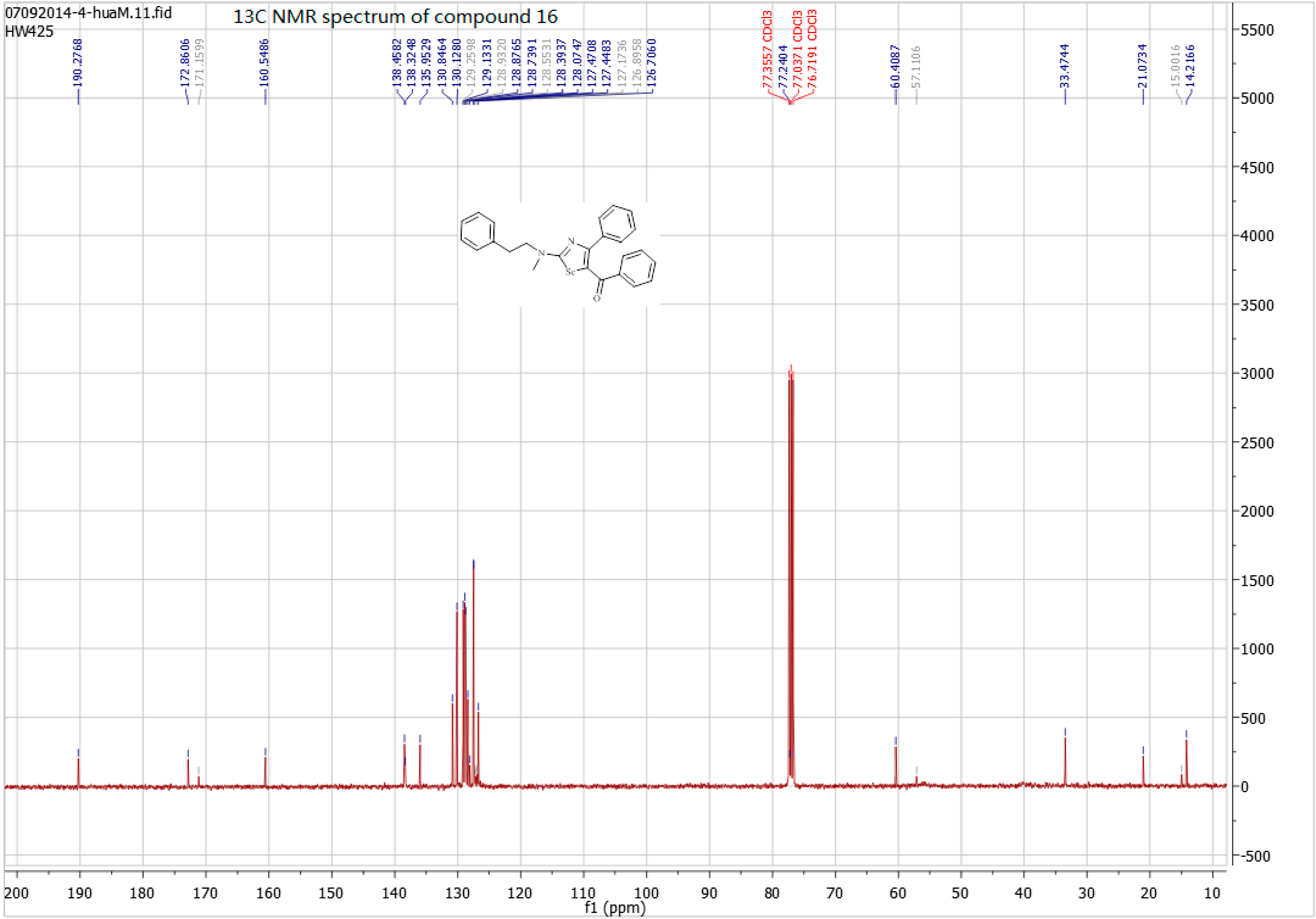
07092014-4-huaM.11.fid
HW425
13C NMR spectrum of compound 16
190.2768
172.8606
171.1599
160.5486
138.4582
138.3248
135.9529
130.8464
130.1280
129.2598
129.1331
128.9320
128.8765
128.7391
128.5531
128.3937
128.0747
127.4708
127.4483
127.1736
126.8958
126.7060
77.3557 CDCl3
77.2404
77.0371 CDCl3
76.7191 CDCl3
60.4087
57.1106
33.4744
21.0734
15.0016
14.2166
f1 (ppm)
